# A Conformal Wearable Ultrasound Bioelectronics for Mechanotherapy Reprogramming of Fibroblast Plasticity via Wnt‐FGF10 Axis to Overcome Fibrotic Healing in Urethral Regeneration

**DOI:** 10.1002/advs.202600037

**Published:** 2026-02-16

**Authors:** Mingming Yu, Xingxuan Zhang, Huan Zhang, Jun Wang, Fang Chen, Xiaojun Cai, Yichen Huang, Yuanyi Zheng

**Affiliations:** ^1^ Department of Ultrasound in Medicine Shanghai Sixth People's Hospital Affiliated to Shanghai Jiao Tong University School of Medicine Shanghai P. R. China; ^2^ Shanghai Key Laboratory of Neuro‐Ultrasound for Diagnosis and Treatment Shanghai P. R. China; ^3^ Department of Urology Shanghai Children's Hospital, School of Medicine Shanghai Jiao Tong University Shanghai P. R. China

**Keywords:** fibroblast reprogramming, fibrosis, low‐intensity pulsed ultrasound, mechanotherapy, urethral regeneration, wearable bioelectronics, Wnt–FGF10 axis

## Abstract

Postoperative fibrotic complications persistently challenge urethral reconstruction due to dysregulated stromal repair and poor graft integration. Conventional rigid ultrasound transducers lack anatomical conformity on dynamic tissues, limiting therapeutic efficacy. Here, we introduce a conformal, wearable low‐intensity pulsed ultrasound (LIPUS) bioelectronic system that overcomes these constraints via controlled adhesion printing and liquid metal interconnects. This low‐cost (<$20), flexible device ensures stable acoustic coupling and programmable mechanostimulation while maintaining mechanical integrity and biocompatibility under repeated use. In a rabbit model of full‐thickness urethral defect, LIPUS significantly improves luminal patency and urinary flow function, promotes angiogenesis and elastic fiber regeneration, while suppressing collagen deposition and pro‐inflammatory macrophage infiltration. Integrated multi‐omics and single‐nucleus RNA sequencing reveal that LIPUS activates the Wnt pathway to drive fibroblast differentiation into a terminally differentiated FGF10^+^ subset (FB3), which engages in regenerative crosstalk with mural cells via FGF10‐FGFR2b signaling and calcium dynamics. Wnt inhibition abrogates this process, confirming mechanistic specificity. This study bridges flexible bioelectronics with deep mechanobiology, providing new insights into how mechanical stimulation can redirect fibroblast fate and override pathological fibrosis, offering a scalable therapeutic framework for fibrotic disorders.

## Graphical abstract

Fibrotic scarring persistently challenges urethral reconstruction. This study introduces a conformal, wearable ultrasound bioelectronic system that overcomes this by reprogramming fibroblast fate. The device activates a developmental Wnt–FGF10 axis, driving fibroblasts toward a regenerative FGF10^+^ phenotype that promotes angiogenesis and matrix remodeling while suppressing collagen deposition, thereby redirecting healing away from fibrosis and toward functional tissue regeneration.

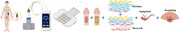

## 1 Introduction

Urethral reconstruction remains one of the most formidable challenges in contemporary urologic surgery, with postoperative fibrotic complications—such as urethral strictures, recurrent fistulas, and tissue contractures—representing a major impediment to functional recovery and long‐term patient satisfaction [1, 2]. Despite significant advances in microsurgical techniques, tissue engineering approaches, and regenerative medicine strategies, the regenerative outcomes are often suboptimal due to dysregulated stromal repair processes, excessive and disorganized extracellular matrix (ECM) deposition, and poor graft‐host tissue integration [3, 4]. Central to this pathological remodeling cascade is the remarkable plasticity of fibroblasts, which, in response to complex mechanical forces, inflammatory cytokine signaling, and local microenvironmental cues, can dynamically adopt either a pro‐regenerative (synthetic) phenotype or a pro‐fibrotic (contractile) phenotype through phenotypic switching mechanisms [5]. Current therapeutic strategies to modulate fibroblast behavior—including direct growth factor delivery, systemic or topical anti‐fibrotic agents, and various biomaterial scaffolds—are frequently limited by their inherently short biological half‐lives, non‐specific off‐target effects, and inadequate spatiotemporal control over therapeutic delivery [3, 6, 7, 8]. Consequently, there exists an urgent unmet clinical need for developing innovative, non‐invasive, precisely tunable, and highly patient‐compliant therapeutic interventions that can selectively guide cellular responses toward constructive tissue regeneration while effectively suppressing pathological fibrotic remodeling through targeted molecular or biophysical modulation [9, 10].

Low‐intensity pulsed ultrasound (LIPUS) has emerged as a promising non‐invasive biophysical modality for enhancing tissue repair and regeneration [11]. It operates by delivering localized mechanical energy in the form of acoustic pressure waves, which can effectively modulate a range of cellular behaviors including proliferation, migration, differentiation, and cytokine secretion [12]. These mechanobiological effects contribute to accelerated healing of injuries, reduced inflammation, and improved functional recovery across various soft and hard tissues [13, 14]. However, conventional LIPUS systems rely on rigid and bulky piezoelectric transducers, which exhibit significant limitations in clinical practice [15]. Their inherent lack of flexibility results in poor anatomical conformity, particularly over curved and dynamic body regions. This leads to inconsistent acoustic coupling, energy loss, and variable treatment efficacy [16]. Moreover, patients are often required to remain stationary during therapy, limiting mobility and comfort, which is a critical drawback for long‐term or home‐based treatments. These issues are especially pronounced in anatomically complex and curvilinear structures such as the penis, where stable device placement and uniform energy delivery are particularly challenging [17]. Despite recent advancements in flexible bioelectronics and wearable sensor technologies, which have enabled soft, stretchable, and skin‐conformable electronic devices for continuous health monitoring [18, 19], the integration of such innovations with ultrasound‐based mechanotherapy remains an underdeveloped area [15]. Moreover, existing flexible ultrasound systems are often designed for relatively static or superficial tissues, lacking the mechanical compliance and anatomical adaptability required for sustained therapy on highly curved and dynamic organs such as the penis. Bridging this technology gap could unlock new possibilities for developing personalized, adaptive, and patient‐friendly LIPUS systems with broad translational potential.

Here, we report the development of a conformal, wearable LIPUS bioelectronic system that overcomes the limitations of conventional rigid transducers and previous flexible designs by enabling consistent and targeted mechanostimulation on dynamic penile tissues. Fabricated via controlled adhesion printing and liquid metal interconnects, this low‐cost and highly flexible device achieves stable acoustic coupling and programmable operation, while demonstrating robust mechanical durability and biocompatibility. Using a rabbit model of full‐thickness urethral defect, we demonstrate that LIPUS treatment significantly enhances luminal patency and restores urinary flow function, promotes angiogenesis and elastic fiber regeneration, and suppresses collagen deposition and pro‐inflammatory macrophage infiltration. Beyond these functional improvements, we employed integrated multi‐omics and single‐nucleus RNA sequencing to unravel the underlying mechanobiological mechanisms. We identified that LIPUS activates the Wnt signaling pathway to drive fibroblast differentiation into a terminally differentiated FGF10^+^ subpopulation (FB3). This specialized phenotype engages in pro‐regenerative crosstalk with mural cells (MC4) via FGF10‐FGFR2b paracrine signaling, further integrating calcium dynamics to potentiate tissue remodeling. Crucially, Wnt pathway inhibition abrogated FB3 expansion and functional recovery, confirming mechanistic specificity. This work bridges the gap between flexible ultrasound bioelectronics and deep mechanobiological insight, establishing a non‐invasive strategy to redirect fibroblast plasticity by reactivating an embryonic Wnt–FGF10 morphogenetic program. By doing so, we override the default fibrotic healing trajectory and promote functional urethral regeneration. Our study not only presents a scalable and patient‐friendly approach for urethral reconstruction but also provides a broader framework for mechanotherapy in fibrotic disorders, highlighting the potential of wearable bioelectronics to decode and harness endogenous regenerative pathways.

## 2 Results and Discussion

### 2.1 A Conformable Wearable LIPUS Bioelectronic System for Anatomically Adaptive and Sustained Mechanotherapy

**FIGURE 1:**
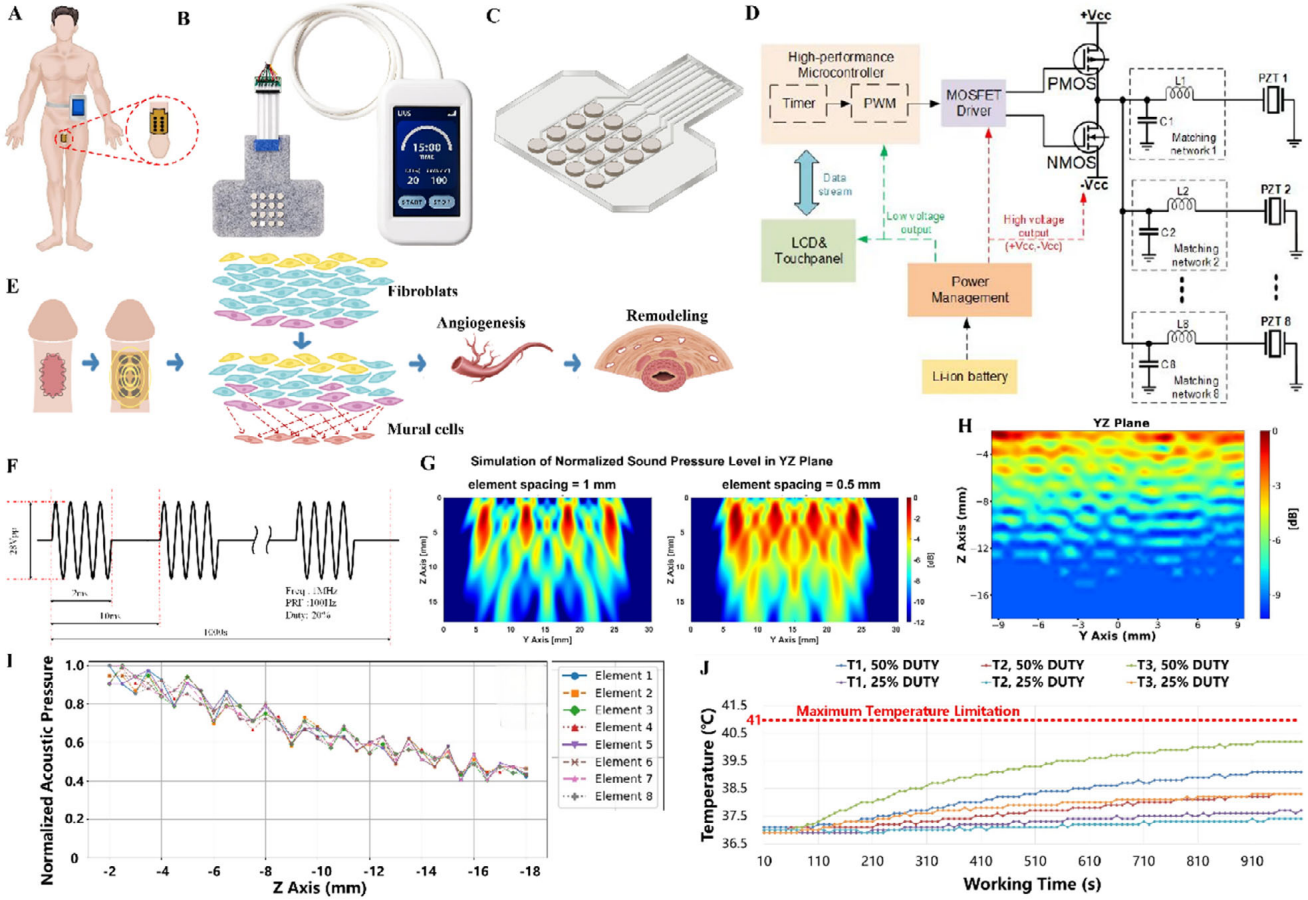
A wearable low‐intensity pulsed ultrasound (LIPUS) device for targeted urethral therapy. (A) Schematic of the wearable LIPUS system designed for conformal penile application. (B) The device comprises a miniaturized control unit and a flexible ultrasonic array patch. (C) The patch integrates sixteen PZT‐5 piezoelectric transducers (5 mm diameter, 4 × 4 array), encapsulated in biocompatible Ecoflex silicone and interconnected via liquid metal through a controllable adhesive printing process. (D) H‐bridge circuit schematic driving the transducers. (E) Mechanobiological mechanism of LIPUS‐enhanced tissue remodeling via intercellular signaling. (F) Representative waveform of LIPUS therapy (1 MHz, 200 mW/cm^2^, 20% duty cycle). (G) Simulated normalized acoustic fields for multi‐element transducers with spacing of 0.5 and 1 mm. (H) Hydrophone‐measured pressure distribution in the YZ plane, confirming effective penetration depths. (I) Elemental output consistency. (J) Thermal safety profile, with temperature elevation stabilized below 41°C under operational limits.

Conventional ultrasound therapy employs rigid, bulky transducers that demonstrate poor anatomical conformity on curved and dynamic tissues [20]. This leads to inconsistent acoustic coupling, energy loss, and limited therapeutic efficacy, especially in complex anatomical regions like the penis [21]. This mismatch between device rigidity and tissue dynamics represents a long‐standing barrier to the clinical translation of ultrasound‐based mechanotherapy [22]. To overcome these limitations, we engineered a conformal, wearable LIPUS system that enables targeted and sustained mechanostimulation for urethral regeneration (Figure 1A). By integrating a soft, stretchable ultrasound array with a miniaturized, programmable control unit (Figure 1B), our system provides a non‐invasive, patient‐friendly, and anatomically adaptive solution for personalized mechanotherapy. This design addresses engineering and clinical challenges within a unified platform. The wearable patch was manufactured via a controlled adhesion printing (CAP) process and low‐melting‐point liquid metal interconnects. This approach imparts exceptional mechanical compliance, including reversible stretchability of 130% to 140%, while maintaining stable conformal contact with biological surfaces. This design ensures consistent energy delivery even under tissue deformation, a critical advancement over existing rigid systems. With a unit cost below $20, which is substantially lower than that of commercial systems (exceeding $5000), the device also establishes a scalable and accessible platform for widespread clinical translation, thereby overcoming the economic barriers typically associated with advanced bioelectronic therapies.

At the core of the therapeutic patch is a 4 × 4 array of 16 PZT‐5 piezoelectric elements (5 mm diameter, 2 mm thickness), individually encapsulated in biocompatible Ecoflex silicone to ensure mechanical robustness and electrical isolation (Figure 1B,C). This configuration not only preserves flexibility but also prevents direct tissue exposure, minimizing the risk of adverse biological reactions. The arrangement and spacing of the elements can be customized to accommodate anatomical variability, enabling patient‐specific dosing. This represents a key innovation for personalized regenerative medicine. A compact control unit, driven by an H‐bridge circuit and powered by a rechargeable lithium battery, allows real‐time adjustment of pulse parameters via an intuitive touch interface (Figure 1D), thereby facilitating precise and localized acoustic stimulation to activate growth factor cascades and promote tissue repair (Figure 1E).

For practical application, the device was operated at a pulse duty cycle of 20% and a repetition rate of 100 Hz (Figure 1F). Hydrophone measurements confirmed compliance with international safety standards (IEC 60601‐2‐5), with spatial peak temporal average (Ispta) and spatial average temporal average (Isata) intensities of 520 and 200 mW/cm^2^, respectively, and a mechanical index (MI) of 0.23. Acoustic field simulations further evaluated the influence of element spacing on field uniformity and device flexibility (Figure 1G). A kerf distance of 5.5 mm was identified as optimal, balancing acoustic homogeneity with mechanical pliability. Needle hydrophone tests validated effective acoustic pressure (>80% of maximum) across a therapeutic depth of 2–10 mm, suitable for targeting the rabbit urethra (Figure 1H). Moreover, the transducers exhibited high output consistency, with less than 10% variation in axial sound intensity under uniform excitation (Figure 1I), ensuring reproducible treatment delivery. Thermal safety assessments in a tissue‐mimicking phantom revealed a maximum temperature rise to 43°C at 0.5 cm depth under extreme conditions (28 Vpp, 50% duty cycle, 1000 s), well within the safe operating threshold and confirming biocompatibility under typical use parameters (Figure 1J) [23].

To comprehensively evaluate the durability of the wearable LIPUS device under prolonged and repeated use, we performed systematic mechanical, electrical, and functional characterization (Figure S1). The conformability of the flexible patch was first quantified using 3D printed cylindrical phantoms with diameters ranging from 25 to 10 mm, confirming its ability to maintain stable, gap‐free contact on surfaces mimicking a spectrum of anatomical curvatures (Figure S1A). Mechanical robustness was evaluated via cyclic tensile testing. The device endured 500 stretching cycles at 15% strain without visible damage, demonstrating resilience beyond the levels associated with typical skin deformation. Even under an extreme 30% strain for 500 cycles, only minor, localized micro cracks were observed at the PZT silicone encapsulation interface, with no element detachment or functional compromise (Figure S1B). Electrical stability, a prerequisite for consistent acoustic output, was verified by measuring the impedance and phase angle of the transducer array before and after 500 cycles of combined stretching and bending. The results showed negligible variation in both parameters, confirming preserved electroacoustic performance after substantial mechanical fatigue (Figure S1C). Critically, functional efficacy in a realistic application context was highlighted by comparing the coupling performance of our conformal array with that of a conventional rigid probe of equivalent active area. In contrast to rigid probes, which are inherently susceptible to geometric mismatch and unstable acoustic coupling on curved tissues, the flexible, circumferentially adhering array provides intimate and uniform contact. This stability mitigates the substantial interfacial energy loss and erratic stimulation patterns characteristic of rigid systems (Figure S1D). This design effectively minimizes pre‐tissue energy attenuation and delivers a more spatially consistent and reproducible acoustic field, which is paramount for reliable mechanotherapy on dynamic anatomical sites like the penis.

To further validate the biocompatibility and safety of the wearable LIPUS system, we performed both in vitro and in vivo assessments. In vitro cytotoxicity testing using human foreskin derived fibroblasts showed >90% cell viability following 15 min LIPUS exposure or incubation with device extracts (Figure S2). In vivo, the flexible patch was adhered to human skin with medical grade silicone tape for 24 h of continuous wear, and no signs of irritation, erythema, or adverse tissue response were observed (Figure S3). Moreover, in the rabbit urethral regeneration model, repeated device application over 14 days did not induce local inflammation or tissue damage, as confirmed histologically. These findings collectively indicate that the device materials, adhesive interface, and acoustic output are well tolerated, supporting its biosafety for prolonged wearable use.

Collectively, by synergistically integrating soft electronics with programmable acoustic output, we have developed a conformal wearable ultrasound bioelectronic system that transcends the inherent limitations of conventional rigid platforms through innovative material engineering and advanced microfabrication techniques. This robust, biocompatible, and cost‐efficient solution establishes a versatile therapeutic platform not only for urethral regeneration but potentially for a broad spectrum of fibrotic and regenerative disorders, marking a significant step forward in the convergence of flexible bioelectronics and precision mechanotherapy.

### 2.2 Enhanced Urethral Regeneration via Wearable LIPUS: Functional and Structural Recovery Beyond Fibrotic Constraints

**FIGURE 2:**
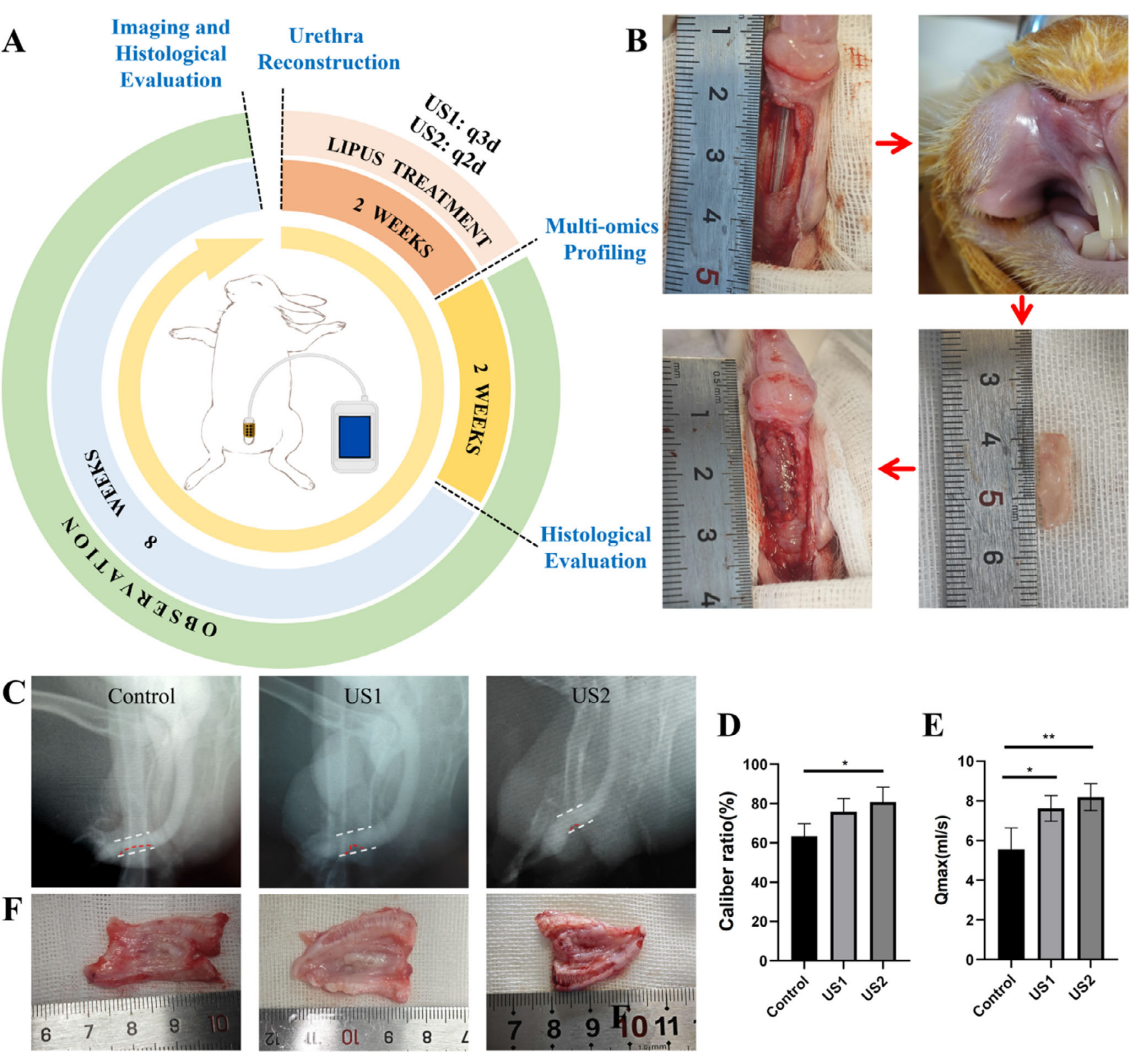
In vivo efficacy of wearable LIPUS in urethral repair. (A) Experimental timeline in a rabbit urethral defect model (US1: treated by LIPUS every 3 days; US2: treated by LIPUS every 2 days; all other parameters identical). (B) Surgical workflow for autologous oral mucosa graft implantation. (C,D) Retrograde urethrography and quantitative luminal patency at 12 weeks, showing superior outcomes in LIPUS‐treated groups. (E) The maximum urinary flow rate (Qmax) was significantly higher in LIPUS‐treated groups compared to the control group. (F) Luminal surface morphology at 12 weeks, with mild irregularity across groups. Data are mean ± SD (n = 4 animals per group). Statistical significance was determined by one‐way ANOVA followed by Tukey's multiple comparisons test (^*^ *p* < 0.05, ^**^ *p* < 0.01).

Despite advances in urethroplasty and tissue engineering, postoperative fibrotic remodeling remains a major impediment to functional urethral regeneration, often leading to strictures and graft failure [1, 8]. While LIPUS has shown promise in modulating penile pathologies through mechanobiological pathways, its clinical translation has been hampered by the anatomical incompatibility of conventional rigid transducers [20, 21, 24]. Here, we demonstrate that our conformal, wearable LIPUS system overcomes these limitations, enabling targeted and sustained mechanostimulation in a dynamic tissue environment. Using a rabbit model of ventral full‐thickness urethral defect, autologous oral mucosa grafts were implanted to bridge the defect (Figure 2A,B). Then the rabbits were randomized into three groups: a control group and two LIPUS‐treated groups differing solely in treatment frequency, designated as US1 (treated every 3 days) and US2 (treated every 2 days). All other therapeutic parameters (frequency: 1 MHz, intensity: 200 mW/cm^2^, duty cycle: 20%, duration: 10 min per session) were identical between the two treated groups. The wearable probe, circumferentially adhered to the penile shaft, delivered consistent acoustic stimulation to the ventral urethra without impeding mobility or causing device‐related complications. All animals survived the study period without severe adverse events such as stricture or fistula, underscoring the safety and biocompatibility of the device. Retrograde urethrography at 12 weeks revealed significantly enhanced luminal patency in US1 and US2 groups, with the US2 cohort outperforming both the control and US1 groups (*p* < 0.05, Figure 2C,D). To further evaluate the functional recovery of the regenerated urethra, we assessed urinary flowmetry. The maximum urinary flow rate (Qmax) was significantly higher in LIPUS‐treated groups compared to the control group (*p* < 0.05, Figure 2E), with the US2 cohort showing the most substantial improvement. This urodynamic enhancement aligns with the observed structural improvements, such as increased luminal patency, reduced fibrosis, and enhanced angiogenesis. These findings suggest that LIPUS‐mediated mechanotherapy not only restores tissue architecture but also effectively improves physiological urethral function.

**FIGURE 3:**
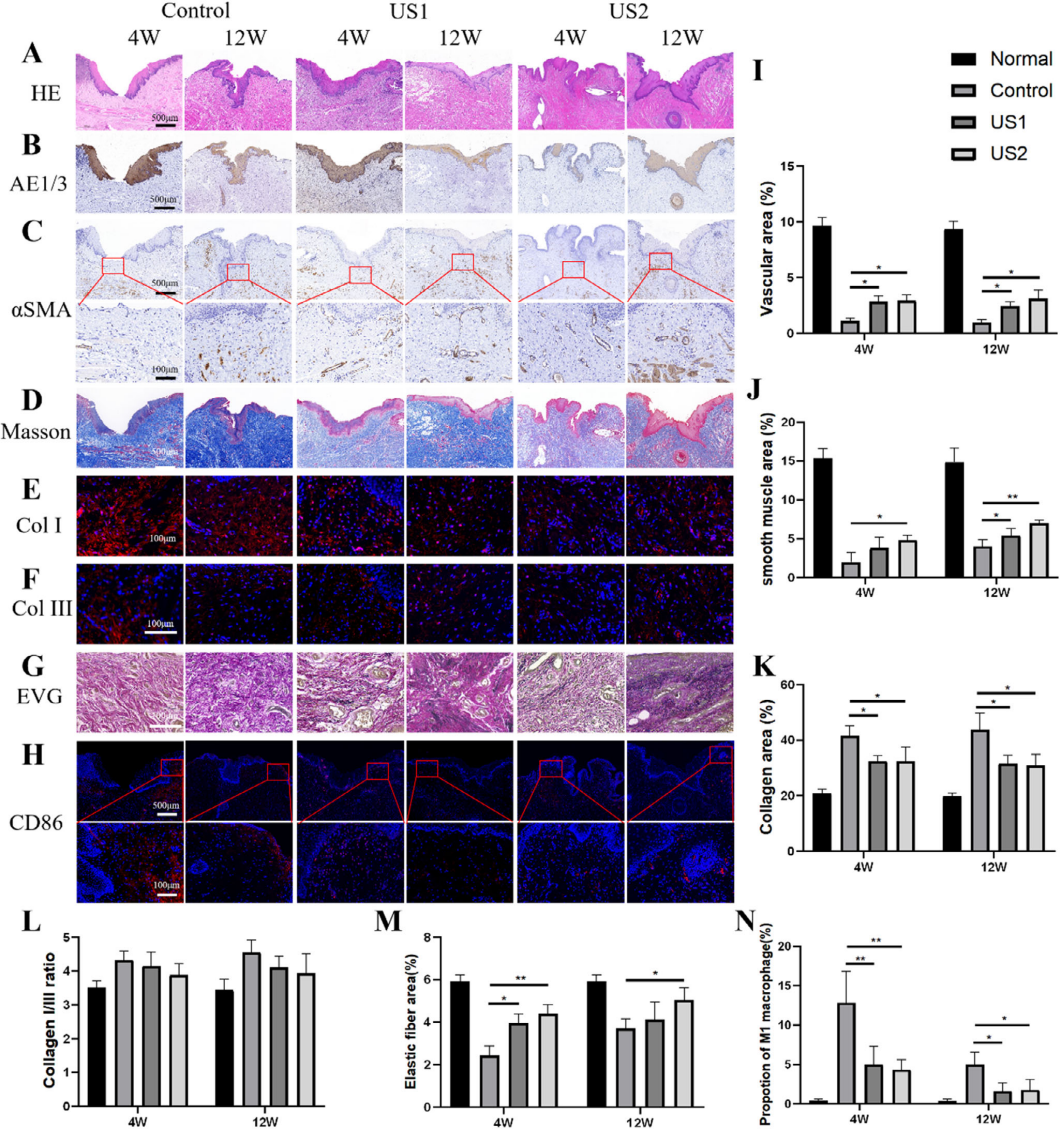
Histomorphometric analysis of LIPUS‐enhanced urethral remodeling. (A‐H) Representative staining: H&E (tissue architecture), AE1/AE3 (epithelium), αSMA (vascular/smooth muscle), Masson's trichrome (collagen), collagen I/III immunofluorescence (matrix composition), EVG (elastic fibers), and CD86^+^ macrophages (inflammation). (I–N) Quantification: LIPUS‐treated groups exhibited increased vascular density (I), smooth muscle content (J), and elastic fibers (M), alongside reduced collagen deposition (K), collagen I/III ratio (L), and CD86^+^ inflammation (N). Quantitative data are mean ± SD (n = 4 animals per group per time point). Statistical analysis was performed using one‐way ANOVA with Tukey's test (^*^ *p* < 0.05, ^**^ *p* < 0.01).

Although mild luminal surface irregularity was observed across all groups (Figure 2F), histological analyses uncovered profound microstructural improvements. H&E staining demonstrated restoration of tissue architecture in the reconstructed segments, with stratified epithelial layers exceeding native urethral thickness, as corroborated by AE1/AE3 immunostaining (Figure 3A,B). αSMA staining revealed distinct spatial heterogeneity in vascular and smooth muscle regeneration, characterized by abundant neovascularization and muscular bundles adjacent to anastomotic sites, contrasting with sparse distribution in central regions. This pattern is indicative of active, progressive tissue remodeling (Figure 3C). Quantitatively, LIPUS‐treated groups exhibited elevated vascular density (*p* < 0.05, Figure 3I) and smooth muscle content (Figure 3J), with progressive enhancement from 4 to 12 weeks, suggesting a sustained pro‐regenerative milieu.

Given the central role of fibrosis in urethral stricture pathogenesis, we focused on collagen deposition and matrix composition. Masson's trichrome staining revealed significantly reduced collagen accumulation in LIPUS‐treated groups compared to controls (*p* < 0.05, Figure 3D,K), though no clear trend was observed between US1 and US2. Given the predominance of collagen types I and III in urethral tissue, we further evaluated the collagen I/III ratio, a recognized indicator of pathological scarring. Treated groups exhibited a 10–15% reduction in this ratio relative to controls (Figure 3E,F,L), indicative of a more regenerative, less fibrotic matrix phenotype. Elastic fiber density, assessed via EVG staining, was significantly elevated in both US1 and US2 groups at 4 weeks, though only US2 maintained this improvement at 12 weeks (*p* < 0.05, Figure 3G,M), highlighting a dose‐dependent benefit of frequent LIPUS application. Inflammation, a dual mediator of regeneration and fibrosis, was evaluated via CD86^+^ macrophage infiltration. LIPUS‐treated groups showed markedly reduced CD86^+^ cell density at anastomotic sites at 4 weeks (*p* < 0.01), with sustained suppression at 12 weeks (*p* < 0.05, Figure 3H,N). While LIPUS is known to modulate inflammatory pathways such as TLRs, NF‐κB, and PI3K/Akt, and to promote M1‐to‐M2 macrophage polarization, we did not observe significant M2 enrichment at 2 weeks after reconstruction according to single‐nucleus RNA sequencing (snRNA‐seq). The observed reduction in macrophages, coupled with the lack of a significant increase in canonical M2 markers at this 2‐week time point, suggests a nuanced immunomodulatory role for LIPUS. It is plausible that the analysis window missed an earlier, transient peak of M2 polarization, which typically occurs during the initial resolution phase of inflammation. By two weeks post‐injury, the immune landscape may have progressed to a later reparative stage. Notably, re‐analysis of our single‐cell data indicated that LIPUS treatment enhanced the overall recruitment of immune cells and enriched for phagocytic subsets expressing high levels of efferocytosis‐related markers (Figure S4). This shift toward a scavenging‐functional phenotype, while distinct from a classical M2 signature, is indicative of an accelerated clearance of tissue debris, a process equally critical for constructive remodeling.

Collectively, these findings establish the wearable LIPUS system as a potent and practical modality for enhancing urethral regeneration. By mitigating fibrosis, suppressing pro‐inflammatory responses, and promoting vascular and muscular restoration, this approach addresses key bottlenecks in urethral repair. The observed dose‐response relationship further highlights the potential for optimizing treatment protocols for clinical translation. More broadly, this work demonstrates how flexible bioelectronics can be leveraged to deliver precise mechanotherapy. This represents a paradigm shift away from static, rigid devices toward adaptive, tissue‐conformal systems capable of guiding complex regenerative processes in dynamic anatomical environments. This represents not only a technical innovation but also a conceptual advance in how we approach tissue engineering and regenerative medicine, bridging the gap between device design and biological response in a clinically relevant context.

### 2.3 Integrated Multi‐Omics Reveals FGF‐Mediated Mechanotransduction as a Central Pathway in Ultrasound‐Driven Repair

**FIGURE 4:**
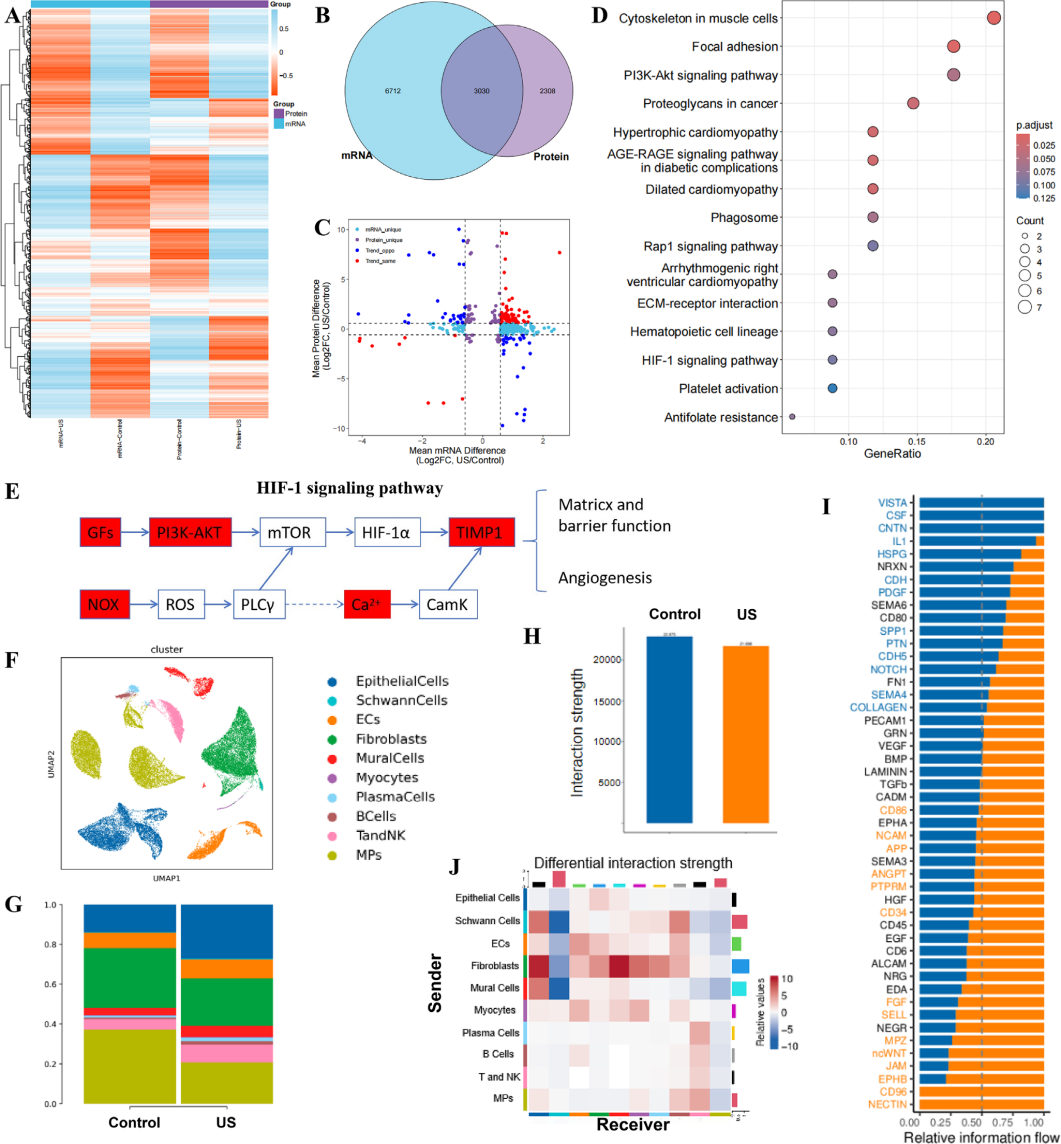
Multi‐omics identifies FGF10‐mediated fibroblast‐mural cell crosstalk as a key mechanism in mechanotransduction. (A–F) Transcriptomics integrated with proteomics reveals the crucial role of growth factor upregulation in mechanotransduction: (A,B) Overlap of 9742 differentially expressed genes (RNA‐seq) and 5338 proteins (proteomics), with 3030 concordantly regulated. (C) Upregulation dominance in both omics layers. (D) KEGG enrichment highlighting PI3K‐Akt, Rap1, and HIF‐1 pathways as central to repair. (E) HIF‐1 pathway activation via upregulated growth factors (FGF, IGF1, HGF, PDGFA), CYBB (NOX), and TIMP‐1, validated by proteomics (Figure S6). (F–J) Single‐cell resolution of FGF‐mediated cellular crosstalk: (F) Uniform Manifold Approximation and Projection (UMAP) visualization of 10 major cell types in urethral tissues. (G) Proportional shifts in cell populations, showing increased epithelial cells (EPCs), mural cells (MCs), and T/NK cells, alongside reduced fibroblasts and mononuclear phagocytes (MPs) in LIPUS‐treated tissues. (H,I) Global interaction strength remained comparable between groups, but FGF signaling exhibited the most pronounced enhancement. (J) Differential interaction analysis identified fibroblasts as primary signal senders and MCs/EPCs as dominant receivers of FGF signaling.

To elucidate the molecular underpinnings of LIPUS‐driven urethral regeneration, we deployed an integrated multi‐omics approach. RNA‐sequencing and proteomic profiling of healing tissues revealed a profound and coordinated response to mechanostimulation, with 9742 differentially expressed genes (DEGs) and 5338 differentially expressed proteins (DEPs) identified. Crucially, 3030 molecules exhibited concordant regulation at both the transcript and protein levels, with a marked bias toward up‐regulation (Figure 4A–C), underscoring the potent, system‐wide activation of reparative programs. This robust dataset allowed us to move beyond correlative observations and pinpoint the core signaling cascades responsible for the observed therapeutic benefits. KEGG pathway analysis decisively implicated the PI3K‐Akt, Rap1, and HIF‐1 signaling pathways as central orchestrators of the LIPUS‐induced repair process (Figure 4D). Complementary Gene Ontology (GO) enrichment highlighted biological processes fundamental to regeneration, including multicellular organism development, system development, and the regulation of cell migration (Figure S5A), which are directly influenced by mechanotransductive forces acting on cellular membranes and cytoskeletal dynamics. Notably, co‐downregulated pathways included ferroptosis, intermediate filament organization, and cholesterol metabolism (Figure S5B). A focused interrogation of the HIF‐1 pathway, a master regulator of angiogenesis and cellular adaptation, revealed a concerted upregulation of its key components. This included upstream growth factors (GFs), the reactive oxygen species generator CYBB (NOX), and the downstream effector TIMP‐1, a critical mediator of ECM remodeling (Figure 4E). Proteomic validation confirmed a significant elevation of specific GFs including FGF, IGF1, HGF, and PDGFA in LIPUS‐treated tissues, with FGF demonstrating the most pronounced increase (Figure S6). The concurrent robust activation of the PI3K‐Akt pathway, a key node within the HIF‐1 axis, establishes a coherent signaling network wherein LIPUS‐stimulated calcium ion flux synergizes with growth factor signaling to drive TIMP1‐mediated vascularization and functional ECM reorganization.

This work provides a critical advance by demonstrating that LIPUS functions not as a mere physical stimulus but as a precise modulator of endogenous signaling landscapes. In contrast to the clinical challenges associated with exogenous growth factor delivery, including short half‐life, instability, and off‐target effects [25, 26], our findings position LIPUS as a sophisticated strategy to spatiotemporally enhance endogenous reparative signals [27]. The dominance of the FGF pathway, a pivotal regulator of angiogenesis and morphogenesis [28], within this multi‐omic signature is particularly significant. It reveals that the therapeutic efficacy of our wearable bioelectronic system stems from its ability to mobilize and amplify endogenous FGF signaling, thereby offering a safer, more controlled, and potentially more efficacious paradigm for regenerative therapy that aligns with the field's shift toward harnessing innate healing mechanisms.

### 2.4 Single‐Cell Transcriptomics Identifies FGF10^+^ Fibroblasts as Orchestrators of Stromal‐Vascular Regenerative Crosstalk

While our multi‐omics data indicated a central role for growth factor upregulation, the cellular sources, targets, and communication networks remained undefined. To deconvolute this complexity, we performed snRNA‐seq, which resolved the urethral tissue into 10 major cell types (Figure 4F). LIPUS treatment induced a significant restructuring of the cellular ecosystem, characterized by an increased prevalence of epithelial cells (EPCs), endothelial cells (ECs), mural cells (MCs), and T/NK cells, alongside a concomitant reduction in fibroblasts (FBs) and mononuclear phagocytes (MPs) (Figure 4G; Table S1). This shift toward a more regenerative cellular composition provided the first cellular‐level evidence of LIPUS efficacy.

Intercellular communication analysis using CellChat revealed that while the global interaction strength was conserved (Figure 4H), the specific signaling patterns were profoundly altered. LIPUS‐treated tissues exhibited a marked amplification of FGF, EGF, and HGF signaling networks (Figure 4I). Strikingly, FGF signaling emerged as the dominant axis, with fibroblasts identified as the primary signal senders and MCs/EPCs as the major receivers (Figure 4J). Ligand‐receptor analysis pinpointed a significant enhancement of FGF7/10‐FGFR2 interactions in treated tissues (*p* < 0.05, Figure S7), indicating a mechanoresponsive reprogramming of fibroblast‐mural cell crosstalk. The functional prioritization of FGF10 over FGF7 can be rationalized by its superior heparan sulfate binding affinity and higher potency for activating the FGFR2b isoform [29], enabling it to more effectively surpass the critical threshold for productive FGFR dimerization necessary for robust tissue repair [30].

Among the 22‐member FGF family with diverse roles [28, 31], our data establish FGF10 as the pivotal mechanoresponsive effector in urethral regeneration. The LIPUS‐induced, fibroblast‐driven FGF10‐FGFR2b signaling axis directly orchestrated MCs‐mediated angiogenesis and EPC‐driven epithelial barrier restoration, operating in synergy with ancillary pathways like VEGF, PDGF, and HGF to coordinate a multifaceted regenerative response (Figure S8). This discovery is transformative, as it shifts the therapeutic focus from administering single exogenous growth factors to leveraging a wearable device to dynamically regulate an entire endogenous signaling network, thereby achieving a more holistic and physiologically relevant pro‐regenerative outcome.

### 2.5 Commitment to a Terminally Differentiated FGF10^+^ Fibroblast Subset Ensures Sustained Secretory and Pro‐Regenerative Functions

**FIGURE 5:**
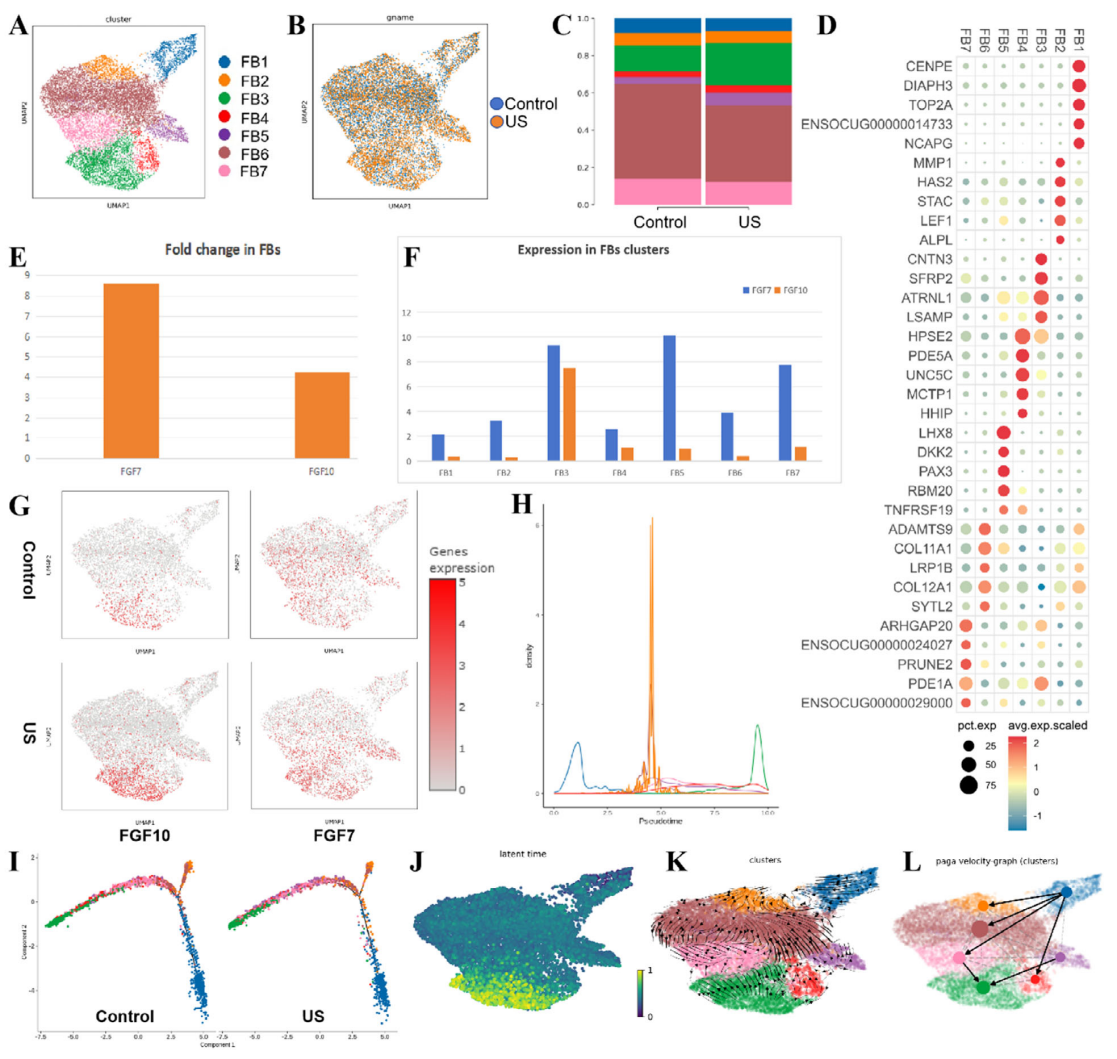
LIPUS‐driven fibroblasts differentiation underpins FGF10‐dominated crosstalk. (A,B) Subclustering analysis identified 7 distinct fibroblast subsets, among which FB3 (FGF10^+^) demonstrated significant expansion in tissues subjected to LIPUS treatment. (C) Proportional distribution of the 7 subclusters. (D) Top marker genes for each subcluster: FB3 uniquely expressed *FGF10*, *COL1A2*, and *POSTN*. (E) Comparative expression of *FGF7* and *FGF10* across cell types, confirming fibroblasts‐specific dominance. (F,G) *FGF10* localized exclusively to FB3, while *FGF7* spanned FB3, FB5, and FB7. (H–J) Pseudotime trajectory, RNA velocity, and latent time analysis positioned FB3 as a terminally differentiated subtype originating from proliferative FB1. (K,L) Lineage reconstruction confirmed LIPUS‐induced FB1‐to‐FB3 differentiation.

To dissect the origin of this potent FGF10 signal, we performed high‐resolution subclustering of fibroblasts, identifying seven distinct subpopulations. This analysis revealed a specific and dramatic 1.6‐fold expansion of the FB3 subset in LIPUS‐treated tissues (Figure 5A–C; Table S2). Transcriptomic profiling defined the FB3 identity by markers including *FGF10*, *COL1A2*, and *POSTN*, with functional enrichment in pathways governing organ morphogenesis and collagen‐rich ECM organization (Figure 5D; Figure S9). A critical finding was the exclusive expression of *FGF10* within the FB3 subpopulation, while *FGF7* was more broadly distributed across FB3, FB5, and FB7 (Figure 5E–G). This establishes FB3 as the principal, dedicated source of FGF10‐driven paracrine signaling, mirroring specialized FGF10^+^ mesenchymal niches known to orchestrate regeneration in organs like the lung and skin [32, 33].

The fate of this pivotal FB3 subset was further illuminated by pseudotime trajectory, RNA velocity, and lineage reconstruction analyses. These computational approaches consistently traced the origin of FB3 to the proliferative FB1 subcluster and positioned FB3 as a terminal node in the differentiation trajectory (Figure 5H–L). This provides compelling evidence that LIPUS does not merely transiently activate fibroblasts but actively steers their plasticity toward a terminally differentiated, pro‐regenerative phenotype committed to sustained FGF10 secretion. This irreversible commitment is a fundamental breakthrough, as it suggests a mechanism to circumvent the cyclical activation and fibrotic conversion that often plagues healing processes.

Parallel analysis of mural cells revealed a corresponding expansion of the MC4 subcluster in LIPUS‐treated tissues (Figure S10A,B). CellChat analysis mapped the FB3‐derived FGF10‐FGFR2b signaling directly onto MC4 (Figure S10C), which was functionally annotated for smooth muscle contraction and development via markers like *KCNMA1* and *COL4A5* (Figure S10D–F). The co‐upregulation of *KCNMA1* (mediating BKCa channel‐dependent hyperpolarization) and *RYR3* (regulating calcium‐induced calcium release) in MC4 suggests a sophisticated dual mechanism: FGF10 signaling, known to trigger intracellular Ca^2+^ release via FGFR‐PLCγ‐IP3 cascades [34, 35], synergizes with intrinsic calcium dynamics to potentiate smooth muscle contractility and transcriptional programs [36]. This mechanochemical integration establishes a feedforward loop that bridges the physical LIPUS stimulus to functional tissue regeneration, offering a novel mechanistic blueprint for how targeted mechanotherapy can calibrate cellular excitability and secretory activity within regenerative niches.

### 2.6 Reactivation of a Developmental Wnt‐FGF10 Axis Redirects Fibroblast Plasticity to Bypass Fibrotic Scarring

**FIGURE 6:**
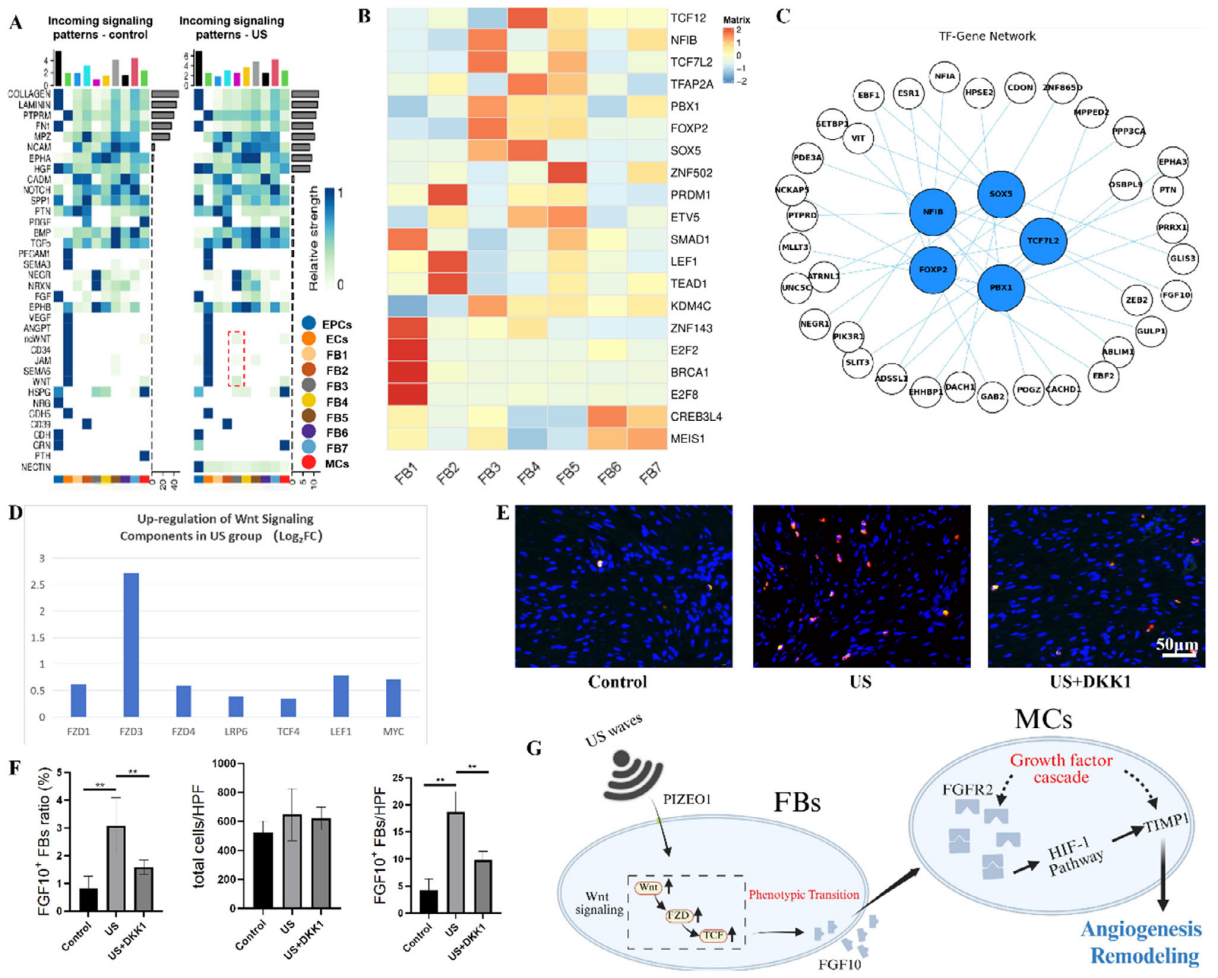
Wnt signaling governs FB3 differentiation and stromal‐vascular crosstalk. (A) Enhanced Wnt/ncWnt signaling to FB3 in LIPUS‐treated tissues. (B) Key transcriptional regulators (*TCF7L2*, *SOX5*, *PBX1*, *KDM4C*) driving FB3 identity. (C) Downstream targets of these transcriptional regulators. (D) Wnt pathway‐upregulated components in the FB3 subpopulation from the US group, including Wnt receptors (FZD1/FZD3/FZD4/LRP6), transcription factors (LEF1/TCF4) and proliferation markers (*MYC*). (E,F) The FISH results demonstrated a significant increase in FGF10^+^ fibroblasts in the US group compared to control, whereas administration of the Wnt inhibitor DKK1 markedly suppressed this effect. DAPI (blue), Collagen I (green, fibroblasts), and FGF10 (red, FB3 subcluster). Scale bar: 50 µm. (G) Mechanistic model: LIPUS activates Wnt signaling to drive FB3 differentiation, enabling FGF10‐mediated MC4 activation and tissue remodeling. Data are mean ± SD (n = 3 per group). Statistical significance was determined by one‐way ANOVA followed by Tukey's multiple comparisons test (^*^ *p* < 0.05, ^**^ *p* < 0.01).

The central question remained: what molecular switch drives the expansion of the regenerative FB3 subset? Our investigation identified the Wnt signaling pathway as the master regulator of this mechano‐driven phenotypic transformation. Transcriptional regulator analysis via pySCENIC pinpointed key drivers of FB3 identity, including *TCF7L2* (a canonical Wnt effector), *SOX5*, *PBX1*, and *KDM4C* (Figure 6A,B), with their downstream networks detailed in Figure 6C. Furthermore, the FB3 subpopulation from LIPUS‐treated tissues displayed a distinct upregulation of Wnt pathway components, including receptors (FZD1, FZD3, FZD4, LRP6), transcription factors (LEF1, TCF4), and the proliferation marker *MYC* (Figure 6D). To functionally validate this discovery, we administered DKK1, a potent Wnt pathway inhibitor, concurrent with LIPUS therapy. Fluorescence in situ hybridization (FISH) confirmed that DKK1 treatment significantly abrogated the LIPUS‐induced expansion of FGF10^+^ fibroblasts (Figure 6E,F). This critical experiment provides direct causal evidence that Wnt activation is indispensable for the ultrasound‐mediated reprogramming of fibroblast fate. To delineate the mechanotransduction profile of fibroblast subpopulations, we quantified pathway module activity across all fibroblast clusters. The LIPUS‐expanded FB3 subset exhibited specific enrichment for canonical Wnt/β‐catenin and FGF10 signaling, whereas classical stiffness sensing pathways including YAP/TAZ, Rho/ROCK, and Integrin/FAK showed no significant activation (Figure S11). This pattern demonstrates that LIPUS‐driven reprogramming selectively engages the Wnt‐FGF10 developmental axis while bypassing the YAP/TAZ‐mediated fibrogenic circuit typically associated with matrix stiffening. These findings culminate in a coherent model (Figure 6G) wherein our conformal wearable LIPUS system reactivates a developmental Wnt‐FGF10 axis, a pathway fundamental to embryonic branching morphogenesis [37, 38], to override the default fibrotic healing trajectory in postnatal tissue. Thereby, this work bridges a critical gap between mechanobiology and developmental biology, demonstrating that mechanical stimuli can be channeled through evolutionarily conserved transcriptional programs to achieve regeneration. While the central role of the Wnt–FGF10 axis is well‐established, the upstream mechanotransductive link, namely how acoustic waves initiate Wnt signaling, warrants further consideration. We hypothesize that LIPUS‐induced membrane deformation may engage key mechanosensors, such as integrin‐focal adhesion complexes (which can activate FAK and PI3K‐Akt signaling to regulate β‐catenin stability) and Piezo1 ion channels (which are ultrasound‐sensitive and can modulate Wnt/β‐catenin via Ca^2+^‐dependent effectors) [39, 40]. These sensors offer plausible molecular bridges between physical stimulus and transcriptional reprogramming. Although our data emphasize the specificity of the Wnt–FGF10 axis, we acknowledge that LIPUS may co‐modulate other mechanoresponsive pathways (e.g., YAP/TAZ), contributing to broader tissue‐adaptive responses. Future studies using real‐time imaging and pathway‐specific perturbations will help delineate their precise interplay.Nevertheless, the synergy between Wnt and FGF signaling, recapitulated here in a therapeutic context, offers a powerful new paradigm: rather than combating fibrosis directly, we can redirect cellular plasticity toward a terminally differentiated, pro‐regenerative state. Our wearable bioelectronic platform thus emerges not merely as a delivery device for ultrasound, but as a non‐invasive tool for precise transcriptional reprogramming, offering a scalable and patient‐friendly blueprint for overcoming pathological fibrosis across a spectrum of regenerative applications.

## 3 Conclusion

Urethral reconstruction continues to be limited by inadequate graft integration and fibrosis, which impair functional recovery. In this study, we developed a conformal, wearable LIPUS system that overcomes the anatomical constraints of conventional rigid transducers, enabling consistent and targeted mechanostimulation of dynamic penile tissues. By integrating controlled adhesion printing and liquid metal interconnects, this low‐cost, flexible device facilitates stable acoustic coupling and programmable operation, thereby providing a practical and patient‐friendly platform for personalized mechanotherapy.

Our results provide new insights into the role of mechanical stimulation in regenerative biology by demonstrating that LIPUS can redirect fibroblast fate through selective activation of the Wnt signaling cascade, driving a terminal differentiation program distinct from conventional mechanotransductive pathways. This process promotes angiogenesis and functional ECM remodeling while suppressing collagen deposition and pro‐inflammatory macrophage infiltration, representing a regenerative trajectory that contrasts sharply with the default fibrotic healing response. Critically, our work highlights the therapeutic potential of selectively modulating fibroblast heterogeneity, a key determinant of repair outcomes. Unlike genetic or pharmacological approaches that target specific subpopulations with limited translational feasibility, LIPUS provides a non‐invasive, spatially tunable, and dose‐controllable means to amplify pro‐regenerative fibroblast phenotypes without systemic toxicity. This strategy aligns with emerging paradigms in scarless healing, wherein precise stromal reprogramming can override pathological remodeling. By reactivating a developmental Wnt‐FGF10 axis, LIPUS effectively repurposes embryonic morphogenetic cues to guide postnatal tissue repair.

The wearable LIPUS system represents a significant engineering innovation, combining soft piezoelectric arrays with miniaturized control modules to enable spatiotemporally precise stimulation of complex anatomical sites. Preclinical validation confirmed significant improvements in vascularization, elastic fiber regeneration, and structural integrity, supporting the system's capacity to enhance biomechanical and functional outcomes. Moreover, the ability to titrate stimulation parameters to modulate Wnt/β‐catenin activation underscores its potential for personalized therapeutic protocols across diverse fibrotic conditions. Beyond urethral repair, this study establishes a broader mechanobiological framework applicable to a range of fibrotic and regenerative disorders. The multimodal mechanism of LIPUS—concurrently targeting vascular insufficiency and ECM dysregulation—suggests compatibility with complementary strategies such as bioactive scaffolds or localized growth factor delivery. Further work is needed to explore the spatiotemporal dynamics of ultrasound‐mediated Wnt activation, including its oscillatory behavior and dose‐dependent patterning of regenerative niches. Techniques such as spatial transcriptomics and in vivo lineage tracing will help elucidate how mechanical dosing guides cellular decision‐making and tissue‐level integration.

Looking toward clinical translation, several practical considerations warrant attention. Anatomical customization could be achieved via patient specific MRI and 3D printing to tailor the transducer array to individual morphology. User‐friendly design, exemplified by an intuitive controller with preprogrammed protocols, would enhance adherence and ease of use in outpatient or home settings. From a regulatory standpoint, the system would likely follow the Class II medical device pathway, necessitating rigorous validation of safety, biocompatibility, and performance consistency. Addressing these engineering and regulatory steps will be essential to transform this wearable mechanotherapy platform into a viable clinical tool for urethral repair and beyond. Future work should also leverage computational modeling of acoustic wave propagation in heterogeneous, layered tissues to develop a predictive framework for optimizing LIPUS parameters (frequency, intensity, duty cycle) based on individual patient anatomy and pathology, moving toward truly personalized dosing regimens. Moreover, while no adverse immune‐related events were observed in this study, future investigations incorporating longitudinal immune profiling, including analysis of T cell subsets and cytokine networks, are warranted to fully characterize the immunomodulatory safety profile of chronic LIPUS application.

In summary, this work not only introduces a scalable and non‐invasive therapeutic platform for urethral reconstruction but also provides fundamental insights into the mechanobiology of fibroblast plasticity. By bridging flexible bioelectronics with deep molecular profiling, we offer a clinically viable strategy to redirect wound healing toward regeneration, with implications for a wide spectrum of fibrotic diseases.

## 4 Experimental Section

### 4.1 Experimental Animals

Adult male New Zealand rabbits weighing 2.5 – 3.0 kg were obtained from the Animal Laboratory of the Shanghai Sixth People's Hospital and raised under clean conditions in individually ventilated cages. All experimental protocols adhered to the ARRIVE guidelines and were approved by the IACUC committee of Shanghai Sixth People's Hospital (approval NO. DWSY2023101). Anesthesia was induced via intravenous administration of 20–30 mg/kg sodium pentobarbital prior to surgical interventions. Euthanasia was performed using a lethal dose of sodium pentobarbital. Animals exhibiting severe complications, including infection, wound dehiscence, or systemic instability, were excluded from the study.

To evaluate the therapeutic efficacy of LIPUS on urethral repair, 24 rabbits were randomized into three groups (n = 8/group): US1 (LIPUS applied every 3 days), US2 (LIPUS applied every 2 days), and control (sham‐treated with an inactive device). Post‐urethroplasty, LIPUS treatment was administered for 14 consecutive days. Histological assessments were conducted at postoperative weeks 4 and 12, with four rabbits per group euthanized at each timepoint.

For molecular mechanism analysis, an additional cohort of six rabbits was randomized into control and US‐treated groups (n = 3/group). The US‐treated group received identical LIPUS parameters as the US2 cohort. Tissue samples were collected at 14 days post‐surgery, immediately following the final LIPUS session, for multi‐omics profiling. To further validate the critical role of Wnt pathway activation in ultrasound‐enhanced urethral repair, another 9 rabbits were divided into control group, US group, and US + DKK1 group (n = 3/group). Tissue samples were collected at 2 weeks post‐operation for FISH analysis to evaluate the expression of FGF10^+^ FBs in the tissues.

### 4.2 Fabrication Protocol for the Wearable LIPUS Device

The fabrication process of the flexible ultrasound transducer array followed a standardized protocol, comprising the following key stages:(1) Piezoelectric Element Preparation: PZT‐5 piezoelectric ceramic material was first cut into cylindrical elements (5 mm diameter) and subsequently polarized. Conductive electrodes were deposited using a standard screen‐printing technique to coat the flanged silver electrodes. (2) Flexible Circuit Fabrication: The flexible ultrasound probe was fabricated based on Controlled Adhesion Printing (CAP) technology, utilizing the SMART 800 PCB printing system from Dream Ink Technology Co., Ltd. (China). First, the circuit pattern was designed in Altium Designer software and imported into the SMART client software. A laser printer was then used to transfer the circuit pattern onto an elastic fabric substrate. Next, the SMART micro‐pressure ink‐leveling system prints liquid metal onto the substrate. (3) Transducer Assembly: The PZT‐5 piezoelectric array was precisely positioned onto the liquid metal circuit to establish electrical connectivity between the liquid metal and the transducer electrodes. A flexible printed circuit (FPC) cable was placed on the opposite side of the liquid metal traces to interface with the host control unit. (4) Encapsulation Process: The assembled sensor array was conformally encapsulated using Ecoflex silicone elastomer. A two‐component encapsulant (A/B ratio = 1:1) was degassed under vacuum (10 min) and then cured at ambient conditions for 4 h, ultimately forming the flexible ultrasound transducer array assembly.

The entire assembly process was conducted at room temperature, avoiding performance degradation caused by high‐temperature soldering. The liquid metal alloy material, developed by Dream Ink Technology, remained in a molten state at 20°C–25°C, with a density of 5500–5800 kg/m^3^ and electrical conductivity of 6.5 ± 0.5 × 10^6^ S/m.

### 4.3 Full‐Thickness Urethra Reconstruction Procedure

Following general anesthesia administration, New Zealand White rabbits were positioned in dorsal recumbency. An 8 Fr transurethral silicone catheter was inserted and maintained in situ. A midline longitudinal incision was made through the peno‐scrotal skin, followed by a controlled longitudinal incision of the ventral tunica albuginea to expose the spongy urethral tissue. A full‐thickness urethral defect measuring approximately 1.5 cm × 0.8 cm was surgically created by precise resection of the ventral corpus spongiosum and the urethral mucosal lining, extending from the coronal sulcus to the penile base. Autologous oral mucosal grafts of appropriate dimensions were harvested and transplanted to reconstruct the urethral defect, with interrupted 6–0 polyglactin sutures utilized for mucosal approximation. Postoperatively, an 8 Fr transurethral polyurethane indwelling catheter was maintained for seven days.

### 4.4 LIPUS Treatment

LIPUS therapy was administered using a programmable wearable device with adjustable irradiation parameters. Under general anesthesia, rabbits were positioned supine with the penis fully exposed. The flexible transducer array was aligned over the ventral reconstructed urethra and circumferentially secured to the penile shaft. Treatment parameters were standardized as follows: frequency of 1 MHz, intensity of 200 mW/cm^2^ and each treatment session lasted for 10 min.

### 4.5 Postoperative Assessment Protocol

At 4 and 12 weeks postoperative, four animals per cohort underwent voiding cystourethrography and urinary flow rate measurement followed by euthanasia under humane conditions for histopathological analysis. Following penile degloving and base amputation, longitudinal incisions were made through the tunica albuginea of the corpora cavernosa from the dorsal surface to expose the ventral urethral reconstruction site. The neourethral tissue was bisected at the midpoint of the anastomotic region. One tissue fragment was formalin‐fixed and paraffin‐embedded for histological evaluation using hematoxylin & eosin (H&E) staining, Masson's trichrome protocol, Elastica van Gieson (EVG) elastin staining, dual immunofluorescence labeling, and immunohistochemical analysis. The contralateral specimen was snap‐frozen in liquid nitrogen for molecular analyses including high‐throughput transcriptomic sequencing and subsequent bioinformatic processing.

### 4.6 RNA‐Sequencing (RNA‐seq)

RNA‐seq analysis was performed following standardized protocols. Total RNA was extracted from biological samples using the Universal RNA Extraction CZ Kit (RNC643, ONREW) following the manufacturer's protocols. Quantitative assessment of RNA integrity was accomplished through fluorometric quantification (Qubit 4.0, Invitrogen) and electrophoretic analysis utilizing formaldehyde‐denaturing agarose gel electrophoresis. Library preparation was conducted using the VVAHTS Universal V8 RNA‐seq Library Prep Kit for Illumina (NR605‐0, Vazyme), with subsequent sequencing performed on the Illumina NovaSeq 6000 platform employing 150‐bp paired‐end sequencing configuration. Comprehensive workflow including mRNA enrichment, library construction, high‐throughput sequencing, and bioinformatics analysis was executed by Shanghai Xu Ran Biotechnology Co., Ltd.

### 4.7 Proteomics

The protein extraction and trypsin digestion protocol involve the following steps: Cell/tissue samples were washed with phosphate‐buffered saline (PBS) to remove blood and debris, then homogenized in lysis buffer (8 m urea, 100 mm Tris‐HCl, pH 8.0) containing protease/phosphatase inhibitors. Samples were sonicated (25% amplitude, 3 s on/3 s off cycles for 1 min), centrifuged at 14 000 × g for 10 min, and supernatants were collected for protein quantification via Bradford assay. For quality verification, 20 µg proteins were separated by SDS‐PAGE (120 V constant voltage, 60 min) and visualized with Coomassie Blue R‐250 staining. For in‐solution digestion, 10 mg proteins were reduced with 10 mm DTT (56°C, 30 min), alkylated with 10 mm IAA (room temperature, dark, 30 min), diluted to 2 m urea with Tris‐HCl, and digested with trypsin (50:1 protein:enzyme ratio) at 37°C for 15–18 h. Reactions were terminated with 0.1% trifluoroacetic acid (TFA), followed by desalting using C18 solid‐phase extraction cartridges. Subsequent nano‐LC‐MS/MS analysis was performed using a nanoElute 2 system (self‐packed C18 columns, 75 min gradient elution at 600 nL/min) coupled to a timsTOF Pro 2 mass spectrometer (positive ion mode, 1.6 kV voltage, 100–1700 m/z scan range, PASEF acquisition mode, 24‐s dynamic exclusion). Raw data were processed with Fragpipe (v21.1) using parameters: trypsin digestion (2 missed cleavages), fixed carbamidomethylation (C), variable modifications (methionine oxidation, N‐terminal acetylation), with mass tolerances of 20 ppm (precursor) and 0.05 Da (fragment).

### 4.8 snRNA‐seq

Mechanically dissociated tissue samples underwent enzymatic digestion followed by sequential processing including filtration through 70/40 µm pore size cell strainers, red blood cell lysis, and optional debris/dead cell removal. Cell viability (>85%) and concentration were quantified using automated fluorescence‐based cytometry (Countstar Rigel S2). Library preparation was performed using the DECODER single cell 3’ transcriptome profiling kit (Dynamic Biosystems), encompassing single‐cell capture, cDNA synthesis, amplification, and subsequent quality control assessments (Qubit 4.0 fluorometer, Agilent 4150 Bioanalyzer). High‐throughput sequencing was conducted on the Illumina NovaSeq 6000 platform employing 150‐bp paired‐end sequencing chemistry.

### 4.9 DKK1 Injection

The DKK1 protein (MCE, USA) was diluted to a working concentration of 2.5 µg/mL. For the US+DKK1 group, 200 µL of the DKK1 working solution was administered via multi‐point injection at the urethral reconstruction site 1 h before each ultrasound treatment session.

### 4.10 Fluorescence In Situ Hybridization (FISH)

Dual FISH was performed using FITC‐labeled Collagen1 (pan‐fibroblast) and Cy3‐labeled FGF10 probes. Co‐localized FITC^+^/Cy3^+^ signals defined FGF10^+^ FBs. RNase‐treated sections confirmed RNA specificity.

### 4.11 Statistical Analyses

The statistical analyses were conducted utilizing SPSS (version 24.0) and GraphPad Prism (version 9.0) software platforms. All quantitative data are expressed as mean ± standard deviation (SD). Prior to statistical testing, the normality of data distribution and homogeneity of variance were rigorously evaluated using appropriate diagnostic procedures. For parametric data meeting criteria for normality and homoscedasticity, independent‐samples *t* test was applied for pairwise comparisons, while one‐way analysis of variance (ANOVA) followed by post–hoc comparisons was employed for evaluating differences across three or more groups. Non‐parametric analyses were performed when data deviated from normal distribution or exhibited heterogeneity of variance, with the Mann‐Whitney *U* test utilized for dichotomous group comparisons and the Kruskal‐Wallis H test implemented for ordinal data involving multiple independent cohorts. Statistical significance was defined as a *p* value < 0.05 following Bonferroni correction for multiple comparisons where applicable.

## Funding

This research was supported by the National Natural Science Foundation of China (82030050, T2394534, 82172074), National Key Research and Development Program 2023YFC2411704, Shanghai Jiao Tong University School of Medicine's “Full‐Time Clinical Research Team” Double Hundred Talents Program SBR2022006, and Innovative research team of high‐level local universities in Shanghai.

## Conflicts of Interest

The authors declare no conflicts of interest.

## Supporting information




**Supporting File**: advs74465‐sup‐0001‐SuppMat.docx.

## Data Availability

The data that support the findings of this study are available from the corresponding author upon reasonable request.
