# Supplementary material for: A Conformal Wearable Ultrasound Bioelectronics for Mechanotherapy Reprogramming of Fibroblast Plasticity via Wnt‐FGF10 Axis to Overcome Fibrotic Healing in Urethral Regeneration
Source: Adv Sci (Weinh). 2026 Feb 16;13(24):e00037. doi: 10.1002/advs.202600037 (PMC13116215; doi:10.1002/advs.202600037)
Supplement: Supplementary file 1 — Supporting File: advs74465‐sup‐0001‐SuppMat.docx. [file ADVS-13-e00037-s001.docx]

Supporting Information

A Conformal Wearable Ultrasound Bioelectronic Device for Mechanotherapy Reprogramming of Fibroblast Plasticity via Wnt-FGF10 Axis to Overcome Fibrotic Healing in Urethral Regeneration

Mingming Yu, Xingxuan Zhang, Huan Zhang, Jun Wang, Fang Chen, Xiaojun Cai*, Yichen Huang*, Yuanyi Zheng*


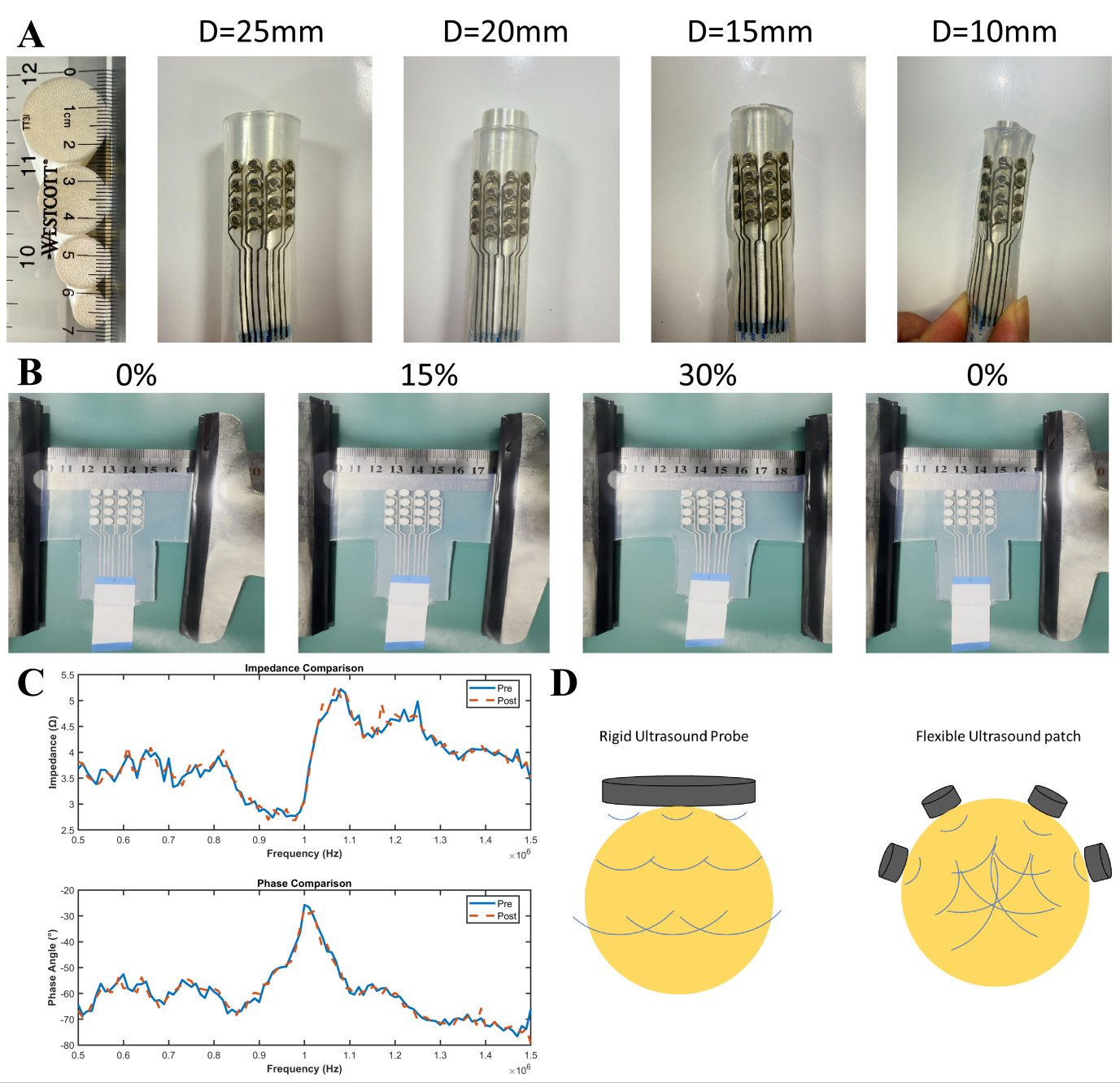


**Figure S1. Mechanical and operational performance of the conformal wearable LIPUS device.** (A) Demonstration of the patch’s conformal adhesion on 3D printed cylinders with diameters ranging from 10 mm to 25 mm. (B) Stretchability test showing that the probe maintains integrity under 15% strain; under extreme 30% strain, minor interfacial cracks appear without functional degradation. (C) Impedance and phase angle measurements before and after 500 cycles of stretching/bending confirm electrical stability and mechanical durability. (D) Comparison of acoustic coupling on curved tissue: a conventional rigid probe (left) exhibits geometric mismatch, leading to inconsistent contact and energy loss; the conformal array (right) ensures uniform coupling, reduced interfacial loss, and repeatable stimulation.

**
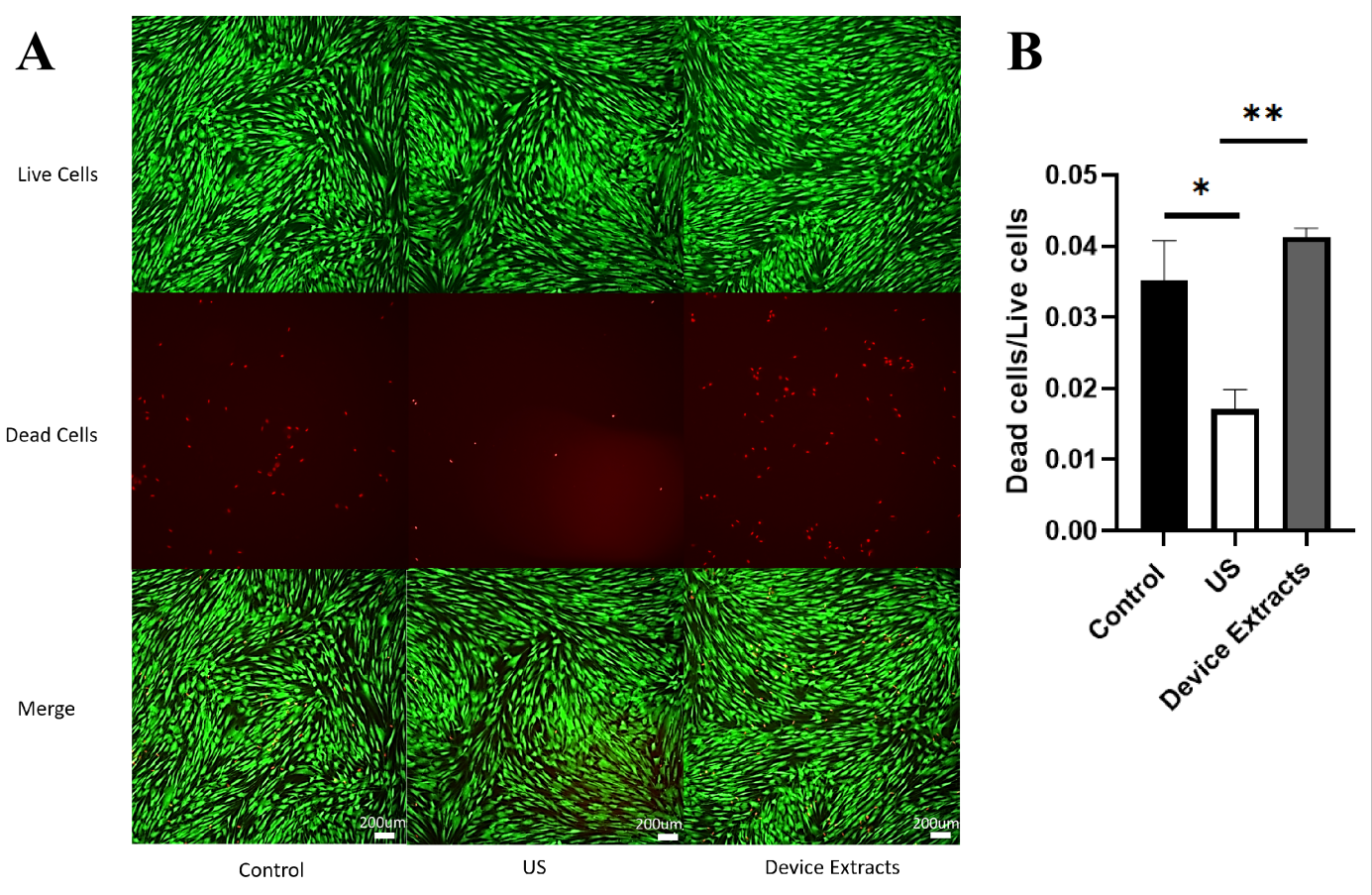
**

**Figure S2. In vitro biocompatibility assessment of the wearable LIPUS system.** (A) Representative live/dead staining (Green: Calcein; Red: AM/PI) of human foreskin derived fibroblasts 24 h after 15 min LIPUS exposure (US group) or incubation with device material extracts (Device Extracts group). Scale bar: 200 µm. (B) Quantitative analysis of cell viability. Data are presented as mean ± SD (n = 6). Notably, the US group exhibited significantly higher cell viability compared to both the control and device extract groups, indicating a potential pro proliferative or cytoprotective effect of the acoustic stimulation itself. No significant cytotoxicity was observed in any group compared to control, and all conditions exceeded the ISO 10993 5 biocompatibility threshold (70% viability, dashed line) (one way ANOVA with Tukey’s post hoc test, **p < 0.01 for US vs. Control and US vs. Extracts).


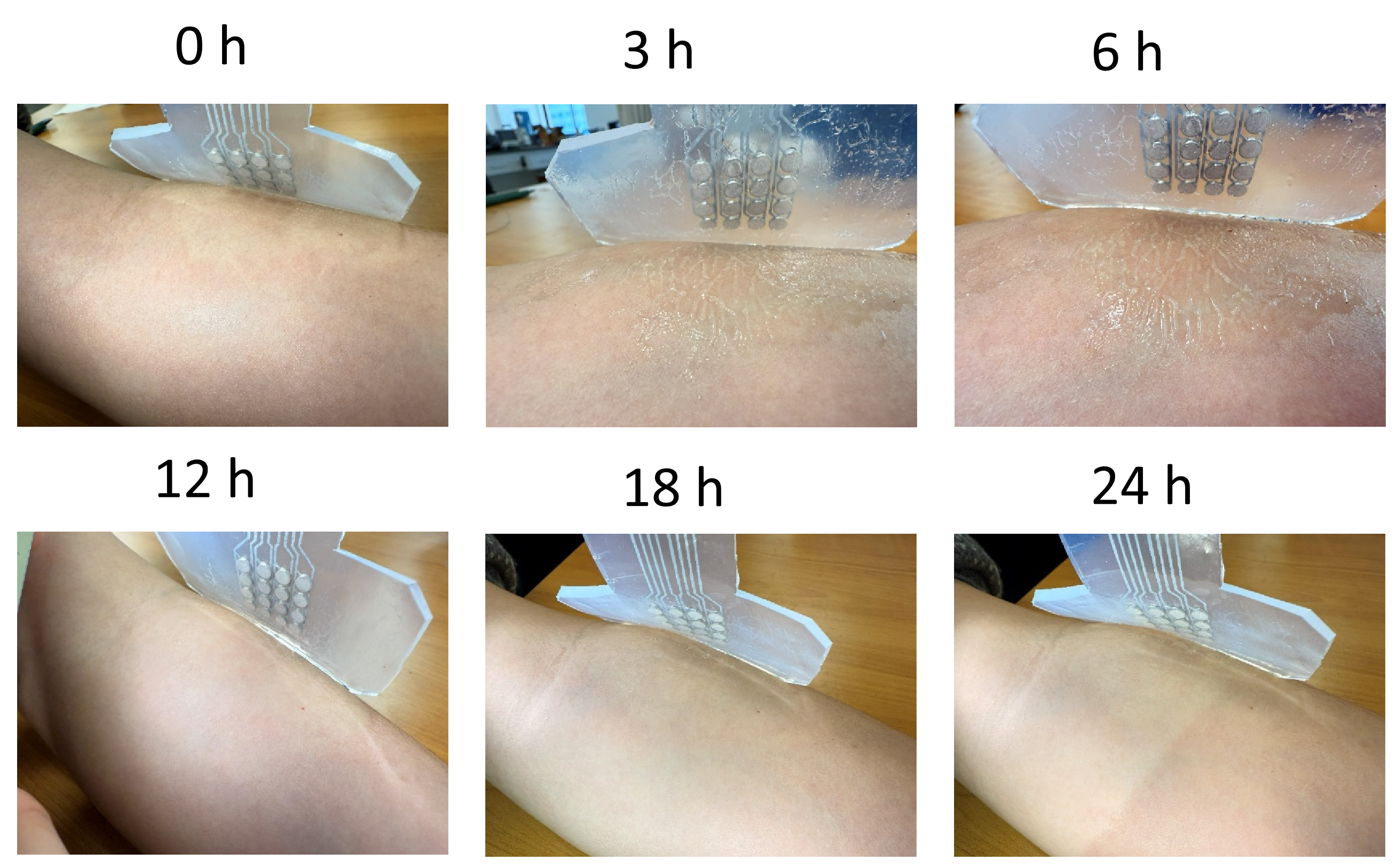


**Figure S3. In vivo skin compatibility assessment of the wearable LIPUS patch.** Photographs of human skin at the device application site were taken at 0, 3, 6, 12, 18, and 24 h during continuous wear with medical‑grade silicone tape. No signs of irritation, erythema, edema, or other adverse tissue reactions were observed throughout the 24‑h period, confirming the short‑term dermal biocompatibility and tolerability of the patch materials and adhesive interface.


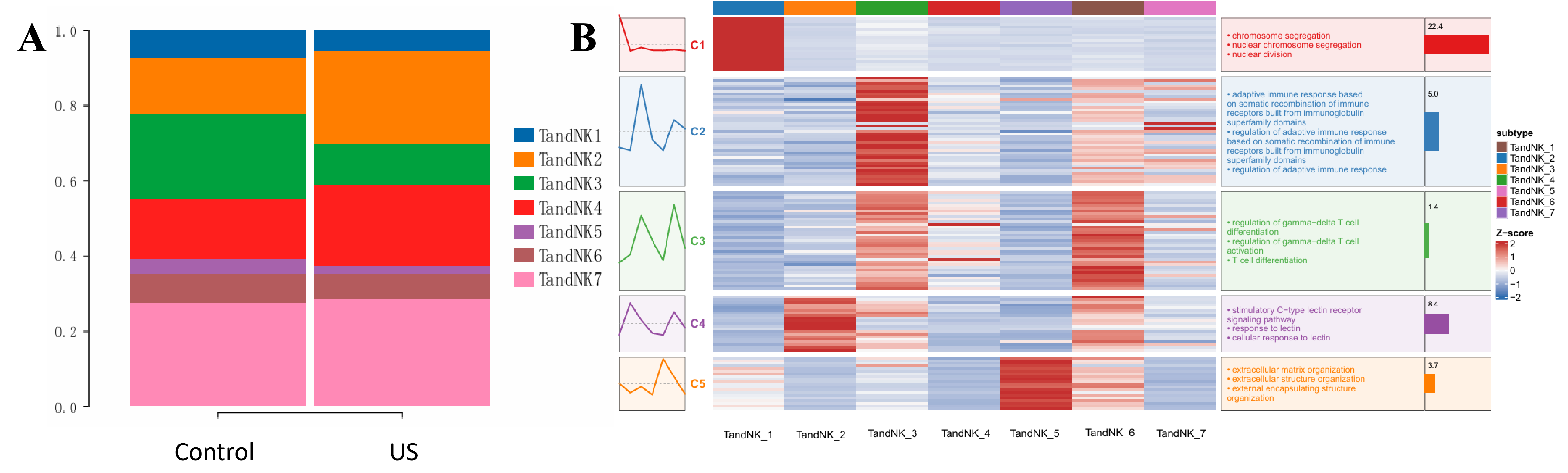


**Figure S4. Compositional and transcriptional analysis of T/NK-cell subpopulations.** (A) Stacked bar plot showing the relative proportions of seven transcriptionally distinct T/NK cell subclusters in control and LIPUS treated tissues. (B) Heatmap of pseudobulk averaged gene expression (Z score per gene) across the seven T/NK subclusters, organized into five co expression modules (C1-C5). Red indicates high expression, blue indicates low expression. Representative Gene Ontology Biological Process (GO BP) terms enriched in each module are listed on the right, with enrichment significance displayed as −log_10_(adjusted P value). Subcluster color coding is consistent between panels.

*
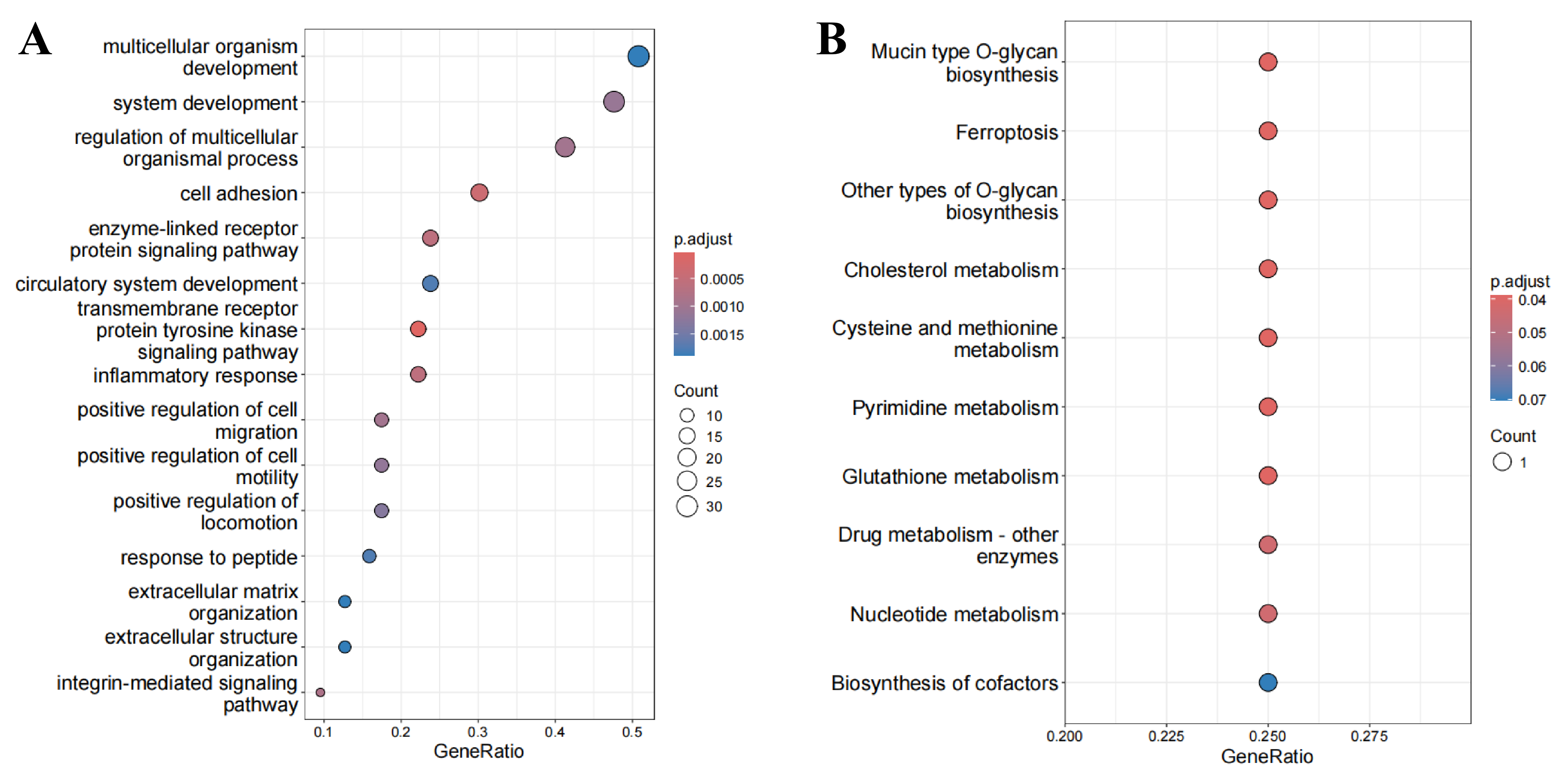
*

**Figure S5. Multi-omics enrichment analysis of concordantly regulated molecules.** (A) GO enrichment of genes and proteins that were co upregulated in LIPUS treated tissues compared to control at 2 weeks post operation. (B) KEGG pathway enrichment of co downregulated molecules in the same comparison.


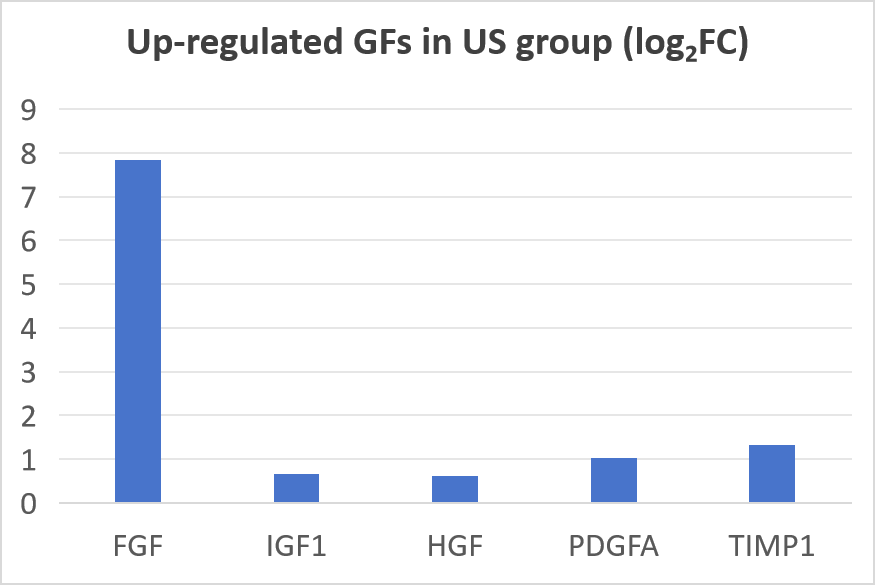


Figure S6. Proteomics-confirmed upregulation of growth factors (FGF, IGF1, HGF, PDGFA, TIMP1).

**
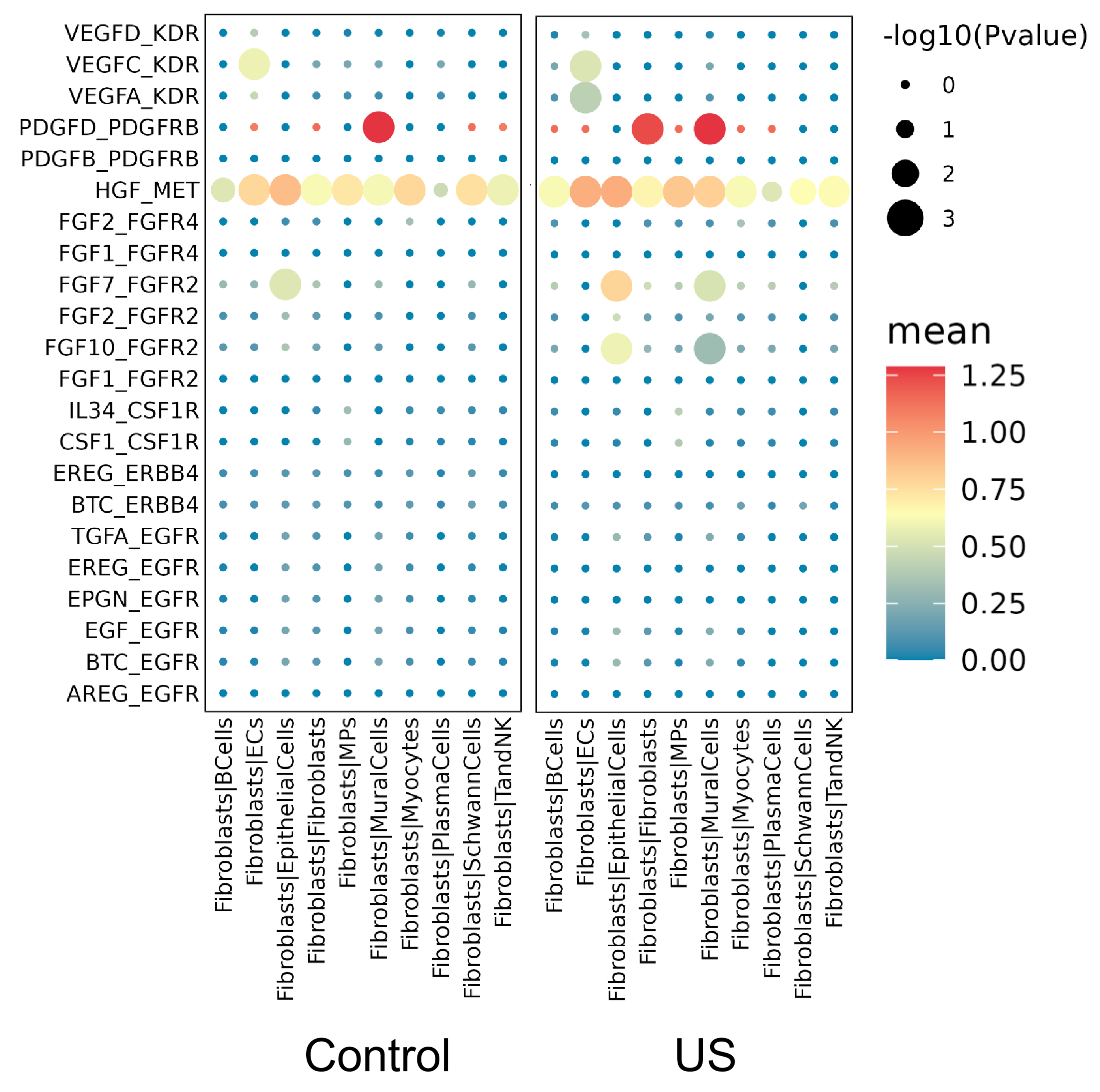
**

**Figure S7. Ligand-receptor pairing revealed amplified FGF7/10-FGFR2 interactions in treated tissues.** FGF7/10-FGFR2 interactions were significantly enhanced in the US group.


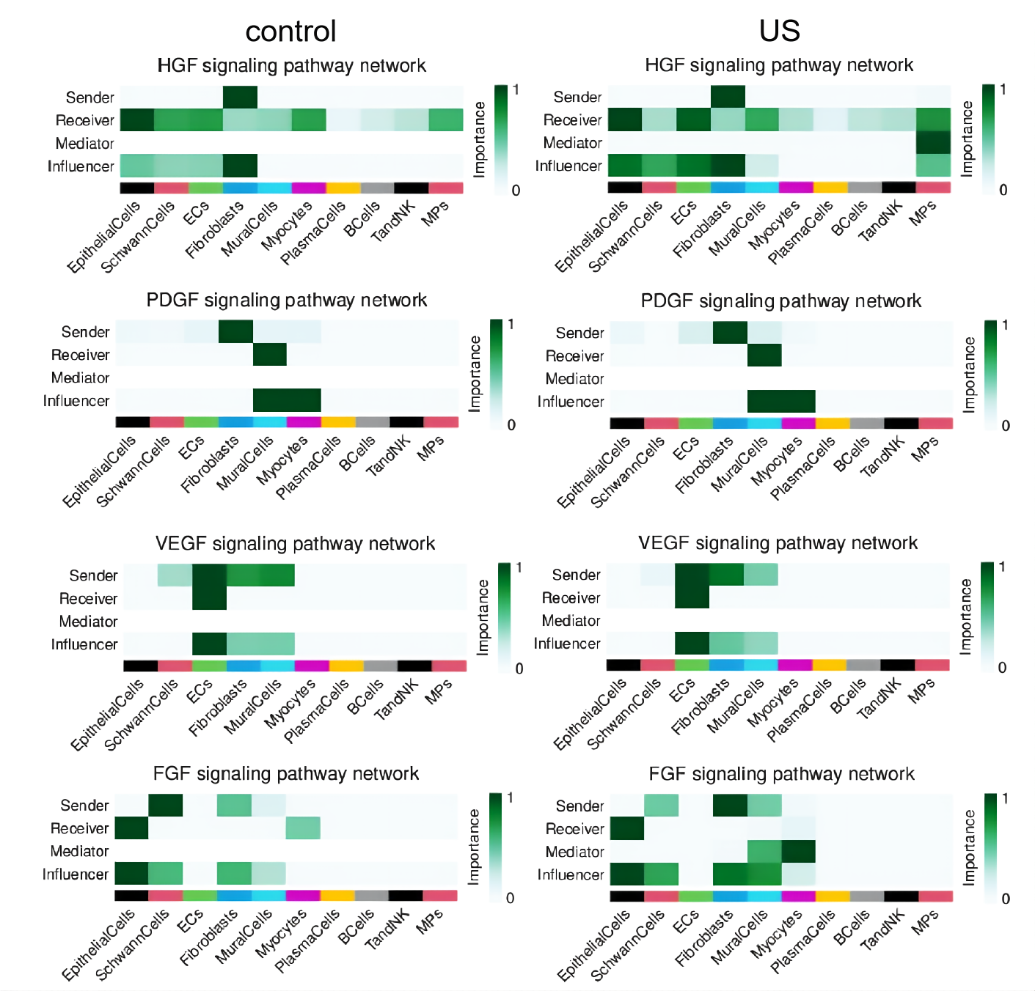


**Figure S8. Heatmaps of GF signaling pathways, highlighting FGF dominance in treated tissues.**


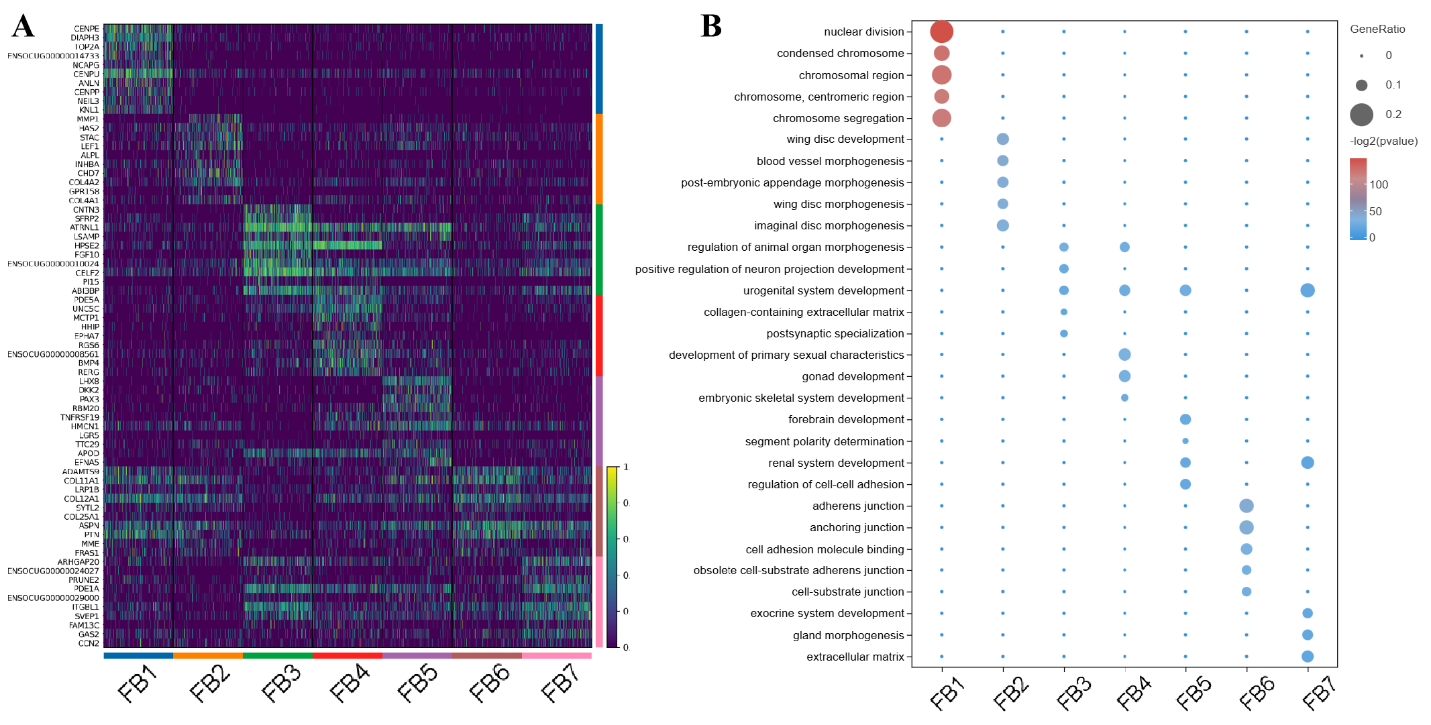


**Figure S9.** The characteristic genes (A) and Gene Ontology (GO) enrichment analysis (B) of the fibroblasts subclusters. The FB3 subcluster exhibited significant associations with biological processes including organ morphogenesis, neuron projection development, urogenital system development, ECM components containing collagen, and postsynaptic specialization.


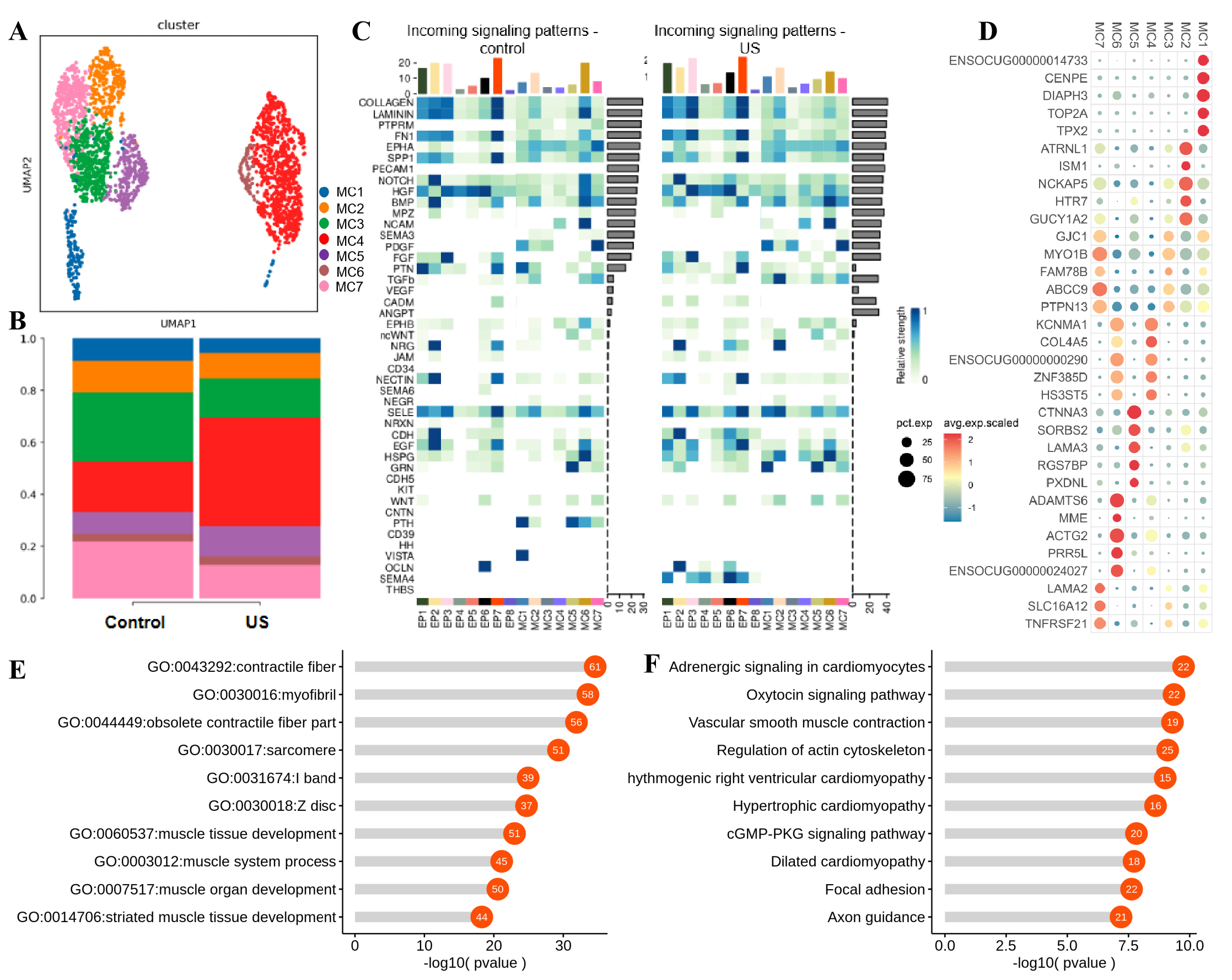


**Figure S10. MC4 subcluster as a functional target of FGF10 signaling.** (A-B) MC subclustering identified seven subsets, with MC4 markedly expanded in LIPUS-treated tissues. (C) CellChat analysis mapped FB3-derived FGF10-FGFR2b signaling to MC4. (D) MC4 markers (*KCNMA1, COL4A5*) implicated in smooth muscle function. (E-F) GO/KEGG enrichment linked MC4 to muscle contraction and development, supported by co-upregulation of *KCNMA1* (BKCa channel) and RYR3 (calcium release).


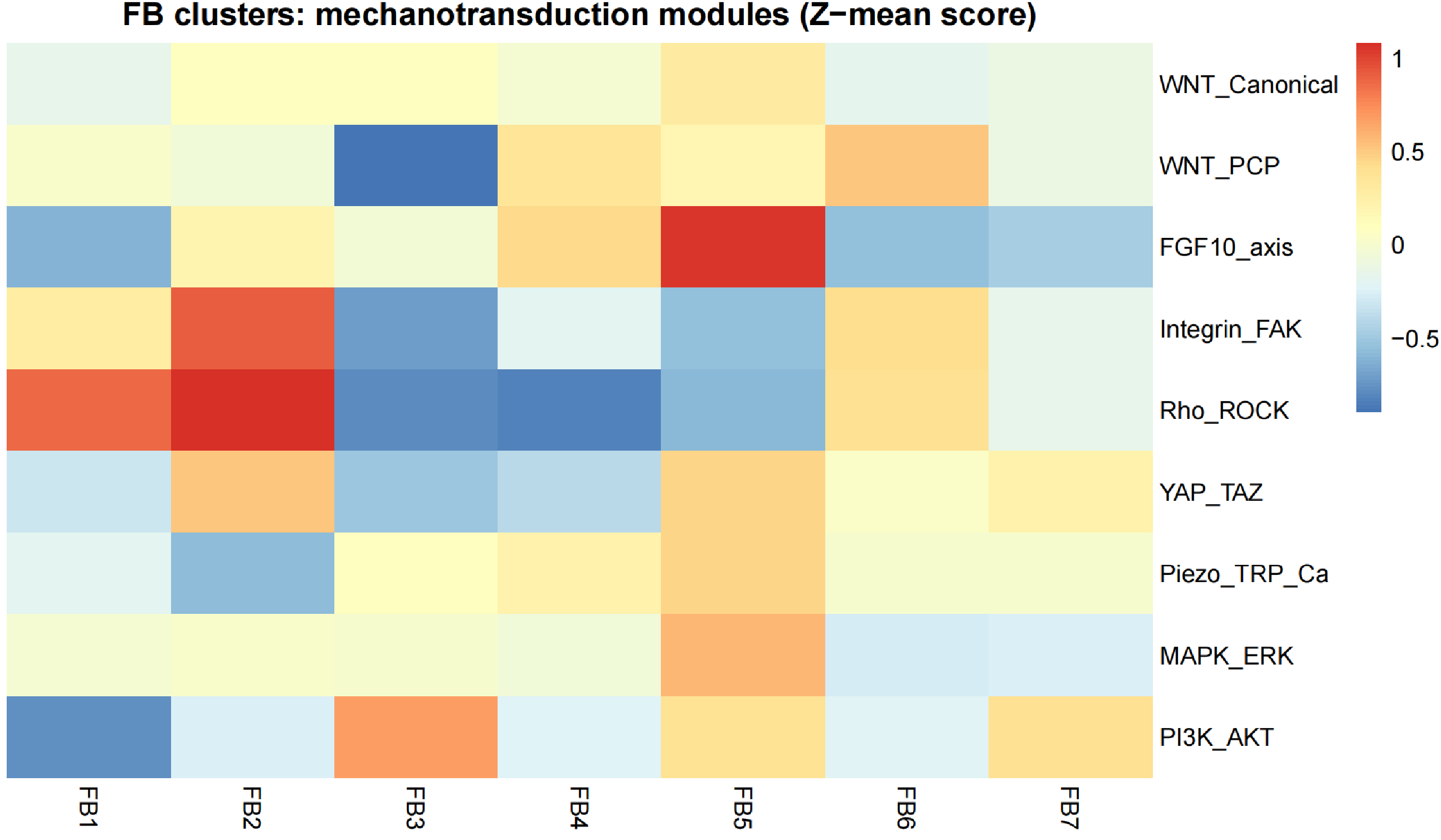


**Figure S11. Mechanotransduction module scores across FB subclusters.** Heatmap showing Z-score-normalized module scores of Wnt/β-catenin signaling (WNT_Canonical), non-canonical Wnt/PCP (WNT_PCP), the FGF10 axis (FGF10_axis), and representative mechanosensitive pathways including Integrin/FAK (Integrin_FAK), Rho/ROCK (Rho_ROCK), YAP/TAZ (YAP_TAZ), Piezo/TRP-mediated Ca^2^⁺ signaling (Piezo_TRP_Ca), MAPK/ERK (MAPK_ERK), and PI3K/AKT (PI3K_AKT) across all FB subclusters.

| **Cell type/Group** | **Control** | **US** |
| --- | --- | --- |
| Epithelial Cells | 3271(13.96%) | 7336(27.22%) |
| Schwann Cells | 46(0.20%) | 88(0.33%) |
| Endothelial Cells | 1808(7.72%) | 2581(9.58%) |
| Fibroblasts | 7050(30.09%) | 6410(23.79%) |
| Mural Cells | 837(3.57%) | 1496(5.55%) |
| Myocytes | 80(0.34%) | 118(0.44%) |
| Plasma Cells | 226(0.96%) | 478(1.77%) |
| B Cells | 184(0.79%) | 448(1.66%) |
| T and NK cells | 1240(5.29%) | 2413(8.95%) |
| MPs | 8691(37.09%) | 5579(20.70%) |
| SUM | 23433 | 26947 |

Table S1. Quantitative distribution and proportional representation of principal cellular subtypes.

| **Cluster/Group** | **Control** | **US** |
| --- | --- | --- |
| FB1 | 542(7.99%) | 436(6.82%) |
| FB2 | 449(6.62%) | 406(6.35%) |
| FB3 | 950(14.00%) | 1451(22.71%) |
| FB4 | 198(2.92%) | 250(3.91%) |
| FB5 | 243(3.58%) | 449(7.03%) |
| FB6 | 3468(51.11%) | 2619(40.99%) |
| FB7 | 935(13.78%) | 779(12.19%) |
| SUM | 6785 | 6390 |

Table S2. Number and percentage of each subcluster in the fibroblasts.
